# CD28‐Targeted Enzyme‐Responsive Conformation‐Switching Peptide Self‐Assembly for Selective T‐Cell Acute Lymphoblastic Leukemia (T‐ALL) Therapy

**DOI:** 10.1002/advs.202520963

**Published:** 2026-04-02

**Authors:** Jun Li, Ziyu Jia, Dekun Li, Zhi‐Wen Hu, Yinghao Ding, Shengyi Zhang, Shengkun Tuo, Zhimou Yang, Huisheng Fu, Ling Wang, Man‐Di Wang

**Affiliations:** ^1^ State Key Laboratory of Medicinal Chemical Biology College of Pharmacy Nankai University Tianjin P. R. China; ^2^ Hemodialysis Center Department of Nephrology Tianjin Medical University General Hospital Tianjin P. R. China

**Keywords:** CD28 receptor, conformation transformation, enzyme‐instructed self‐assembly, peptide self‐assembly, T‐cell acute lymphoblastic leukemia

## Abstract

T‐cell acute lymphoblastic leukemia (T‐ALL) is a highly aggressive hematologic malignancy with limited targeted therapies. CD28, a costimulatory receptor aberrantly overexpressed on T‐ALL cells, presents a promising underexplored therapeutic target. In this study, we developed an enzyme‐responsive self‐assembling peptide, SA^p^‐CD28, designed to target CD28 and undergo receptor‐mediated self‐assembly in the tumor microenvironment. Upon dephosphorylation by overexpressed phosphatases, SA^p^‐CD28 transitions from an α‐helix to a β‐sheet/β‐turn rich structure, facilitating the formation of nanooligomers that engage CD28 and activate cytotoxic pathways. Transcriptomic and biochemical analyses reveal that SA^p^‐CD28 induces a profound dysregulation of CD28 downstream signaling, characterized by the suppression of the PLCγ and Akt pathways. These signaling perturbations lead to oxidative stress and disruption of intracellular calcium homeostasis, resulting in calcium overload, calpain activation, and cytoskeletal collapse. Besides, confocal imaging suggested that the peptide self‐assembly can enter the nucleus and disrupt it. In Jurkat xenograft models, SA^p^‐CD28 demonstrated potent antitumor activity, and its combination with cytarabine resulted in near‐complete tumor suppression, highlighting its potential for T‐ALL treatment. This work introduces a CD28‐targeted, enzyme‐activated nanotherapeutic strategy that synergizes biochemical and mechanical mechanisms to selectively eliminate T‐ALL cells. This multi‐mechanistic tumor‐killing strategy can also be extended to inspire therapeutic approaches for other diseases.

## Graphical abstract

We developed the enzyme‐responsive peptide SA^p^‐CD28 to selectively target CD28‐overexpressing T‐ALL cells. Following phosphatase‐mediated activation, SA^p^‐CD28 undergoes conformational switching and nanooligomerization, resulting in the disruption of CD28 downstream signaling. This includes suppression of the PLCγ and Akt pathways, triggering oxidative stress, calcium overload, cytoskeletal collapse, and necrotic cell death. In vivo, SA^p^‐CD28 effectively inhibited Jurkat xenograft growth and tumor inhibition.

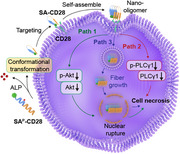

## 1 Introduction

T‐cell acute lymphoblastic leukemia (T‐ALL) is a highly aggressive hematologic malignancy, accounting for approximately 15% of pediatric and 25% of adult acute lymphoblastic leukemia (ALL) cases [1, 2]. Especially, T‐ALL in adults remains associated with a poorer clinical outcome, largely due to a higher risk of relapse and resistance to conventional treatments [3, 4, 5]. Despite advances in chemotherapy and immunotherapy, the treatment of T‐ALL is still complicated by the lack of specific, druggable surface targets. Currently available targeted therapies focus on a few receptors, such as CD3, CD5, and CD7 [6], however, these targets have significant limitations. For instance, CD3‐targeted therapies can cause T‐cell fratricide [7, 8, 9], while CD5 and CD7 therapies can be toxic to normal immune cells [10, 11, 12, 13]. Additionally, the high heterogeneity of T‐ALL complicates the identification of universally accessible targets [14]. This underscores the urgent need to discover new, highly specific targets for the treatment of T‐ALL that can selectively eliminate malignant cells while sparing normal T cells and reducing systemic toxicity.

CD28 is a classical costimulatory receptor that plays a central role in T‐cell activation and downstream signaling. In T‐ALL cells, CD28 is not only aberrantly overexpressed [15, 16, 17, 18] but also structurally accessible on the cell surface, providing an opportunity for therapeutic exploitation [19]. Unlike other immune checkpoint receptors such as PD‐1 and CTLA‐4, which are typically targeted in immunotherapy, CD28 is highly expressed in T‐ALL, whereas CD28 expression is often downregulated or functionally exhausted on normal T cells within the tumor microenvironment [20, 21, 22, 23, 24]. This suggests that CD28 may represent a promising yet underexplored candidate for selective intervention in T‐cell malignancies.

**FIGURE 1:**
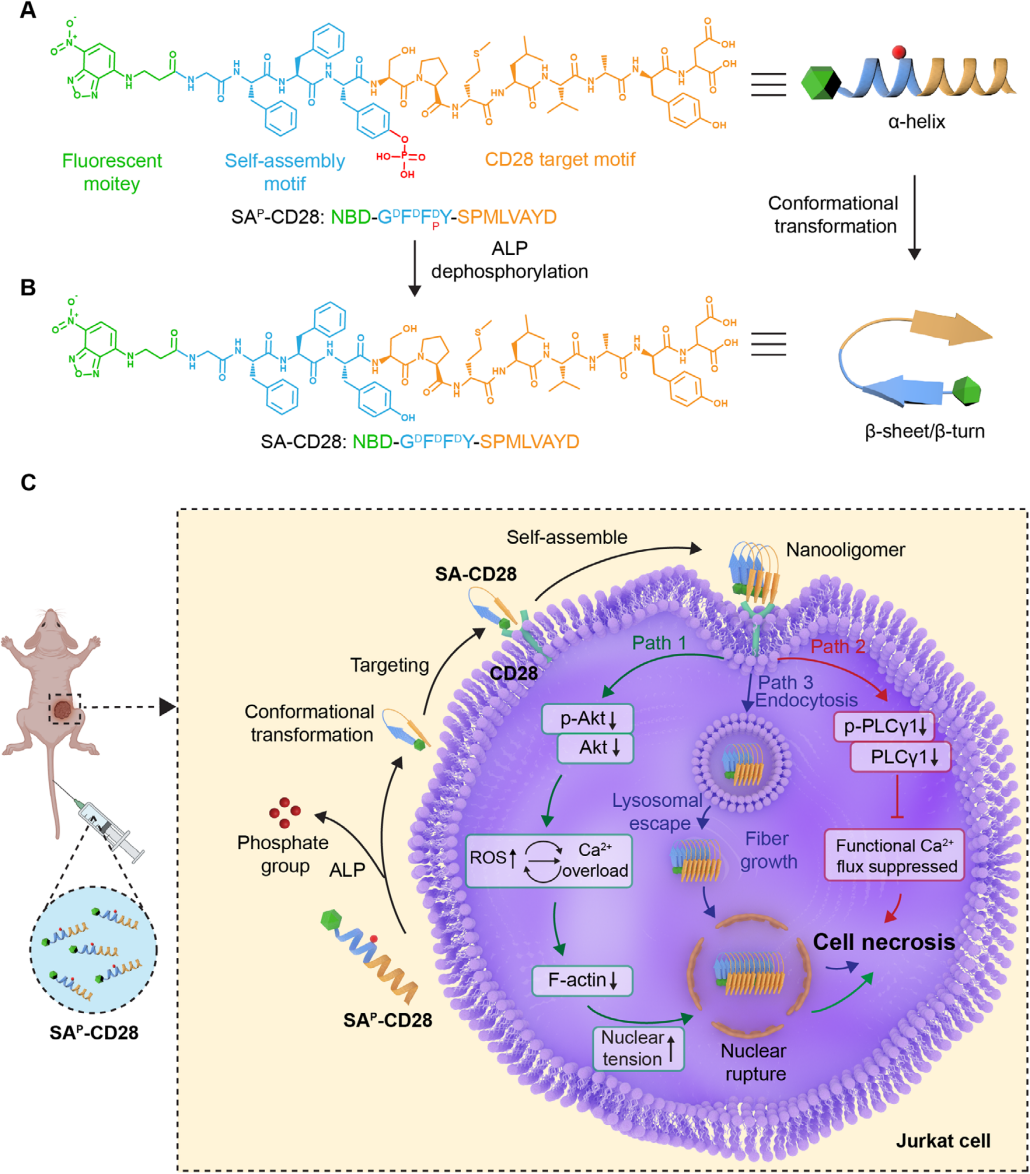
Schematic of peptide molecular design and its tumor‐killing mechanism. (A) Chemical structure and secondary conformation of SA^p^‐CD28. (B) Chemical structure and secondary conformation of SA‐CD28, generated via alkaline phosphatase‐mediated dephosphorylation of SA^p^‐CD28. (C) Schematic illustration of SA^p^‐CD28 inducing nuclear rupture through dysregulation of CD28 downstream signaling and physical damage, and its synergy with the chemotherapeutic agent cytarabine in cancer treatment.

In recent years, enzyme‐instructed intracellular self‐assembly of small molecules and peptides has emerged as a powerful strategy for regulating cellular functions and enhancing therapeutic efficacy through the in situ formation of bioactive nanostructures [25, 26]. Herein, we designed a modular enzyme‐responsive self‐assembling peptide, named SA^p^‐CD28 (NBD‐G^D^F^D^F^D^_p_Y‐SPMLVAYD) (Figure 1A), which can selectively target CD28 and undergo enzyme catalysis and receptor‐mediated in situ self‐assembly. The combination of affecting the related downstream pathways and physical disruption of the nucleus synergistically kills T‐ALL cells. The molecular structure consists of three parts: (1) the CD28‐targeting peptide SPMLVAYD, enabling CD28 receptor‐specific binding [27]; (2) the self‐assembly motif G^D^F^D^F^D^_p_Y, with a phosphorylated tyrosine residue [28, 29]; and (3) the fluorescent moiety NBD, incorporated for molecular tracking and facilitation of assembly. SA^p^‐CD28 exhibits excellent solubility, allowing for intravenous administration and systemic circulation. When it arrives at the tumor site, the molecule is dephosphorylated by phosphatases, which are overexpressed in the tumor microenvironment, resulting in the formation of SA‐CD28 (Figure 1B). This enzymatic cleavage disrupts the hydrophilic‐hydrophobic balance of the peptide molecule, triggering a conformational change from an α‐helix to a β‐sheet/β‐turn structure. This facilitates the in situ assembly of the nanooligomer through engagement with the CD28 receptor.

After self‐assembly, SA‐CD28 nanooligomer engages CD28 and triggers leukemic cells' death by three paths (Figure 1C). mRNA transcriptomic profiling, validated by Western blotting (WB), revealed two principal mechanisms: (1) PLCγ1 pathway (Path 2): total PLCγ1 decreased by 48%, while the ratio of phospho‐PLCγ1 (Tyr783) to total PLCγ1 dropped by 80%, indicating suppression of canonical PLCγ‐mediated calcium signaling; (2) Akt pathway (Path 1): total Akt protein levels were reduced by more than 54%, suggesting inhibition of the PI3K‐Akt survival pathway and disruption of cellular metabolic homeostasis. This metabolic imbalance promotes oxidative stress and reactive oxygen species (ROS) accumulation, which in turn perturbs intracellular calcium homeostasis and induces calcium overload. Elevated ROS and calcium form a self‐amplifying cycle that activates the calcium‐dependent protease calpain, leading to cleavage of cytoskeletal proteins and ultimately triggering necrotic cell death. Confocal imaging further indicated that the oligomers are endocytosed, escape from the lysosomes, and eventually enter the nucleus. Due to the nucleation mechanism of peptide self‐assembly, the nanooligomers continue to elongate after entering the cells, ultimately forming large nanofibers that mechanically disrupt the nucleus (Path 3). In vivo experiments demonstrated that SA^p^‐CD28 exhibited potent antitumor activity in the Jurkat xenograft model, and when combined with cytarabine, achieved near‐complete tumor suppression.

## 2 Results and Discussion

### 2.1 Modular Peptide Design for Phosphorylation‐Dependent Self‐Assembly

First, in terms of molecular design, besides the star molecule SA^p^‐CD28, we also designed three control molecules, named SA‐CD28^p^ (**NBD‐G^D^F^D^F^D^Y‐SPMLVA_p_YD**), which has distinct phosphorylation sites compared to SA^p^‐CD28. SA^p^ (**NBD‐G^D^F^D^F^D^_p_Y**), and CD28^p^ (**NBD‐SPMLVA_p_YD**), each lacking either the assembly motif or targeting sequence. All of the molecules were capped with the fluorescent molecule NBD to ensure real‐time tracking and consistency of the hydrophobic termini. Phosphate groups were used for labeling to ensure the water solubility and enzyme‐responsive properties of the molecules. The strategic integration of D‐amino acids was employed to improve enantiomeric stability and protect against proteolytic degradation [30, 31]. ALP‐mediated dephosphorylation of pY induced controlled supramolecular assembly through charge neutralization and hydrophobic interactions [32, 33]. The chemical structures of all peptides were illustrated in Figure 1 and Figures S1–S4, with detailed synthetic protocols and high‐resolution mass spectrometry (HRMS) characterization provided in the Supporting Information.

**FIGURE 2:**
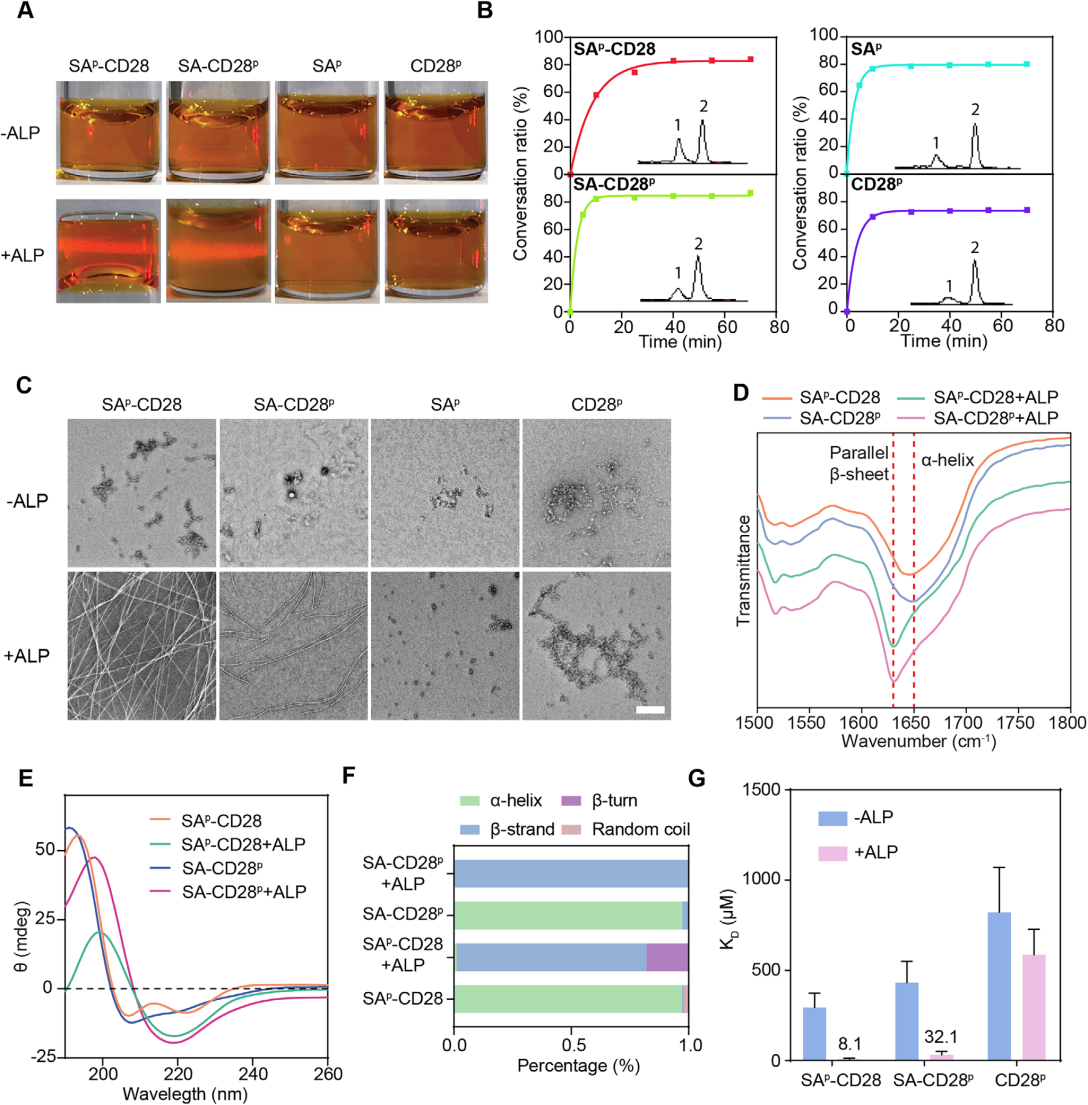
Characterization of the four peptide molecules before and after ALP dephosphorylation reactions. (A) Tyndall effects of the four peptides after incubation without ALP or with ALP for 12 h. (B) Conversion rates of the four peptides incubated with ALP (10 U/mL) over time. The HPLC chromatogram (inset images) shows two peaks, corresponding to the peptides before (peak 1) and after ALP dephosphorylation products (peak 2). (C) Transmission electron microscopy (TEM) images of the indicated peptides incubated without ALP or with ALP for 12 h. Scale bar: 200 nm. (D) FTIR spectra of SA^p^‐CD28 and SA‐CD28^p^ in the presence or absence of ALP (10 U/mL). The dashed lines represent parallel β‐sheets and α‐helices, respectively. (E) CD spectrum of SA^p^‐CD28 and SA‐CD28^p^ in the presence or absence of ALP (10 U/mL). (F) Secondary structure calculation of SA^p^‐CD28 and SA‐CD28^p^ in the presence or absence of ALP (10 U/mL). (G) Microscale thermophoresis (MST) analysis of SA^p^‐CD28, SA‐CD28^p^ and CD28^p^ binding to the recombinant human CD28 proteins before and after ALP dephosphorylation reactions. K_D_ values represent apparent binding affinities calculated from a 16‐point titration. Data represent mean ± SD from three independent biological replicates (*n* = 3).

The in vitro functional characterization of the synthesized peptides began with a comparative analysis of solubility profiles and supramolecular assembly dynamics across four peptide molecules, both before and after catalytic enzymatic treatment. Shown in Figure 2A, all four peptide molecules demonstrated excellent solubility in their pre‐catalytic states, as indicated by the absence of Tyndall scattering, confirming complete molecular dissolution. After ALP treatment (5 U/mL, 12 h), distinct differences emerged: SA^p^‐CD28 formed a mechanically stable colloidal matrix, exhibiting gravitational resistance and a strong Tyndall effect, while SA‐CD28^p^ showed intermediate assembly properties, with detectable light scattering but no macroscopic gelation. In contrast, SA^p^ and CD28^p^ remained in solution with minimal colloidal formation, suggesting impaired assembly capabilities.

Subsequent LC‐MS analysis of dephosphorylation kinetics revealed rapid enzymatic activity across all variants, with approximately 80% phosphate cleavage occurring within 40 min, as evidenced by characteristic hydrophobic shifts in the chromatographic profiles (Figure 2B). Transmission electron microscopy (Figure 2C) revealed phosphorylation‐dependent nanostructural changes: SA^p^‐CD28 transitioned from pre‐catalytic nanorod aggregates to an interpenetrating nanofibrillar network (fiber diameter: 4.15 ± 0.32 nm), while SA‐CD28^p^ reorganized from sparse nanospheres into unidirectionally aligned fibers (6.21 ± 0.45 nm diameter). In contrast, SA^p^ and CD28^p^ exhibited minimal morphological changes. Fourier‐transform infrared (FTIR) spectroscopy (Figure 2D; Figure S6) revealed that both SA^p^‐CD28 and SA‐CD28^p^ exhibited a prominent absorption band at 1645∼1647 cm^−1^, characteristic of the α‐helix structure. Upon dephosphorylation, this peak shifted to 1628∼1631 cm^−1^, indicating the formation of β‐sheet structures [34]. Figure S6 shows that SA^p^ displayed an amide I peak at ∼1637 cm^−1^, corresponding to β‐sheet or random‐coil conformations, which remained unchanged after dephosphorylation. In contrast, CD28^p^ displayed an α‐helical band at 1645∼1647 cm^−1^ that shifted to 1633∼1635 cm^−1^ following phosphatase treatment, indicating an α‐to‐β structural transition. The circular dichroism (CD) curve and Secondary structure calculation analysis are shown in Figure 2E,F, which revealed distinct conformational transitions across all assemblies. Notably, SA^p^‐CD28 underwent a complete α‐helix to β‐sheet/β‐turn conversion (96% reduction in α‐helix content, accompanied by 80.3% β‐sheet formation and 17.9% β‐turn acquisition), while SA‐CD28^p^ displayed near‐total α‐helix elimination (97.3% reduction), transitioning exclusively to β‐strand architecture [35, 36, 37]. The SA^p^ and CD28^p^ structure transformation are shown in Figures S7 and S8. SA^p^ exhibited β‐sheet, dominated conformations both before and after enzymatic treatment, whereas CD28^p^ initially showed α‐helix structures that converted to β‐sheet‐rich structures upon dephosphorylation, consistent with the FTIR observations.

Functional validation through comparative microscale thermophoresis (MST) analysis of pre‐ and post‐enzymatic states demonstrated significant improvements in CD28 binding affinity (Figure 2G; Figures S9 and S10). SA^p^‐CD28 exhibited a dramatic decrease in apparent dissociation constant from 293.8 ± 79.2 µm in the pre‐ALP state to 8.1 ± 5.4 µM after ALP‐triggered assembly, corresponding to a 36.2‐fold enhancement in binding affinity. In comparison, SA‐CD28^p^ showed a more modest 13.4‐fold reduction in apparent K_D_ (from 431.4 ± 118.1 to 32.1 ± 19.7 µM), while CD28^p^ exhibited persistently weak binding (K_D_ > 500 µM) regardless of enzymatic treatment. Given that ALP treatment induces peptide dephosphorylation and promotes multimeric assembly, the measured K_D_ values represent apparent binding affinities reflecting the overall interaction strength between peptide assemblies and CD28. These results suggest that the superior affinity of SA^p^‐CD28 arises from its capacity to couple ALP‐triggered dephosphorylation with structural reorganization into a β‐turn conformation. Previous studies have shown that many peptide ligands adopt turn or loop conformations, particularly β‐turns, at receptor‐binding interfaces [38, 39, 40, 41]. Pre‐stabilizing such β‐turn structures through chemical conformational constraints or β‐turn mimetics can enhance peptide‐protein binding affinity or preserve agonistic activity. Therefore, the β‐turn dominated reorganization observed in SA^p^‐CD28 likely provides a structural basis for its markedly improved CD28 binding and activation capacity.

### 2.2 Specific Cytotoxicity of SA^p^‐CD28 Peptide Molecule Against T‐ALL Cells

**FIGURE 3:**
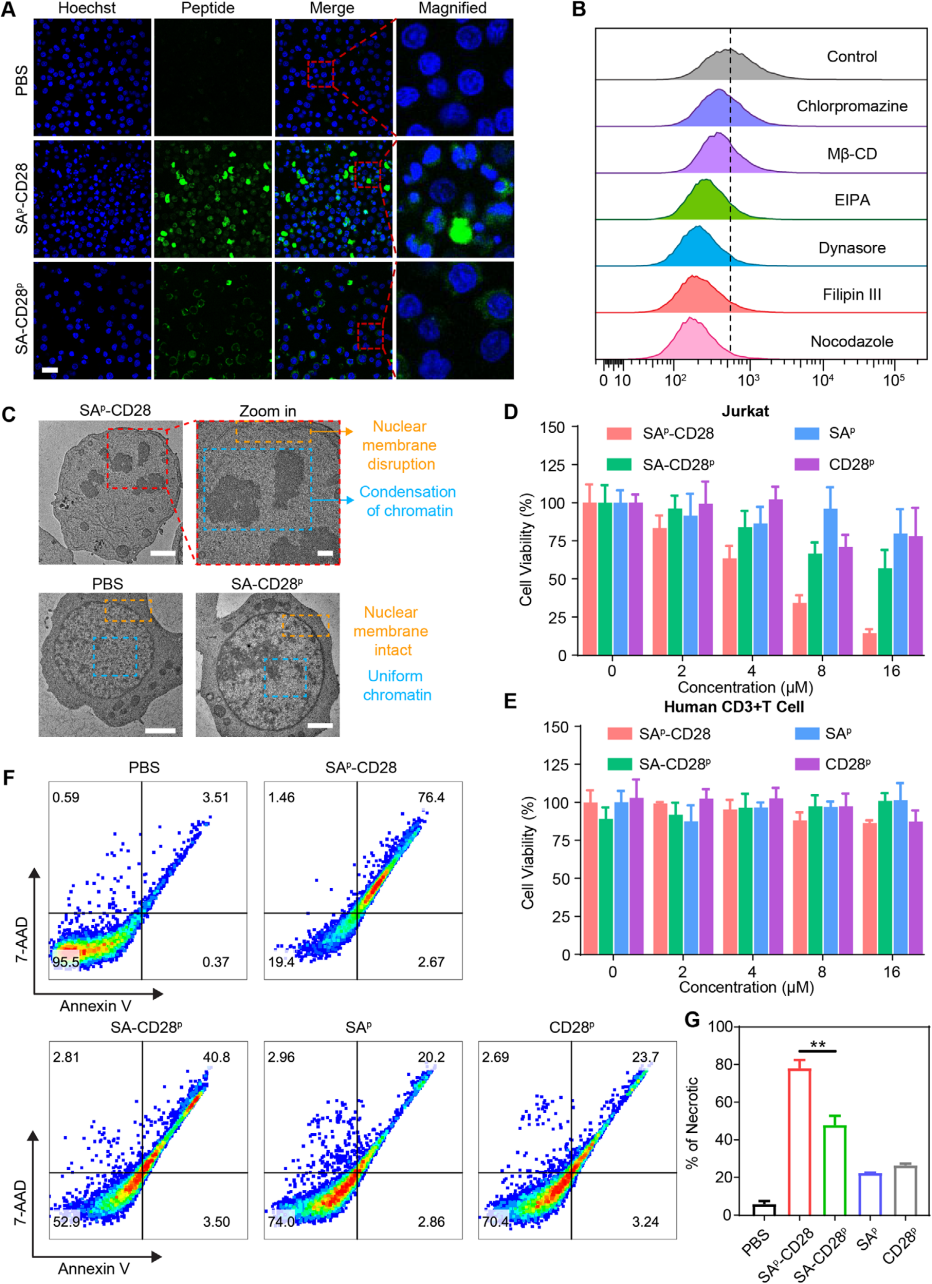
Nuclear damage and apoptosis triggered by peptide self‐assembly at the cellular level. (A) Confocal laser scanning microscopy (CLSM) images of Jurkat cells treated with SA^p^‐CD28 or SA‐CD28^p^. Scale bar: 25 µm. (B) Flow chart of fluorescence intensity of Jurkat cells pretreated with different internalization inhibitors, then incubated with SA^p^‐CD28 for 1 h. (C) Biological electron microscopic (Bio‐EM) imaging of Jurkat cells after the indicated treatments for 8 h. Scale bar: 500 nm. (D, E) Relative cell viabilities of (D) Jurkat cells and (E) human CD3^+^ T cells incubated with different concentrations of the four peptides for 48 h (*n* = 4). (F) Annexin V‐PE/7‐AAD apoptosis detection of Jurkat cells after various treatments for 12 h. (G) Quantitative analysis of apoptosis flow cytometry results. Data are presented as mean ± SD (*n* = 3 independent experiments). Statistical analysis was performed using one‐way ANOVA. ^**^ *p* < 0.01.

To investigate the cellular biological functions of the synthesized peptides, we used ALP‐ and CD28‐high‐expressing Jurkat cells as a model system. Figure S11 shows that Jurkat cells exhibited a high extracellular alkaline phosphatase (ALP) release rate, while Figure S12 confirmed strong surface CD28 expression by flow cytometry. Shown in Figure 3A, co‐incubation of Jurkat cells with 100 µM peptides for 12 h revealed distinct patterns of cellular interaction: SA^p^‐CD28 demonstrated efficient internalization, with a homogeneous intracellular distribution, accompanied by prominent nuclear fragmentation as observed through Hoechst 33342 staining, a phenotype indicative of chromatin disruption. In contrast, SA‐CD28^p^ predominantly localized to the membrane, without nuclear penetration or damage. However, SA^p^ and CD28^p^ showed minimal cellular uptake (≈10%) and no detectable nuclear effects (Figure S13). Quantitative flow cytometry (Figure S14) confirmed similar total cell‐associated levels (>90%) for both SA^p^‐CD28 and SA‐CD28^p^, while confocal microscopy further localized SA^p^‐CD28 to cytoplasmic compartments, suggesting that ALP‐triggered structural reorganization enables intracellular penetration.

Subsequently, we investigated the lysosomal escape of SA^p^‐CD28 at the subcellular level. As shown in Figure S15, after 2 h of treatment, the peptides were highly colocalized with lysosomes. After 4 h, most peptide molecules no longer colocalized with lysosomes and were instead distributed throughout the cytoplasm, indicating that the peptide successfully escaped from the lysosomes. As shown in Figure 3B and Figure S16, treatment with various inhibitors led to a reduction in peptide uptake to varying degrees. Notably, treatment with nocodazole (a β‐tubulin inhibitor), dynasore (a cell‐permeable dynamin inhibitor), and EIPA (a macropinocytosis inhibitor) reduced the internalization efficiency of SA^p^‐CD28 to 37.83%, 43.63%, and 49.00%, respectively. These results indicate that the cellular uptake of SA^p^‐CD28 primarily occurs through a β‐tubulin‐, dynamin‐, and macropinocytosis‐dependent, multi‐protein‐mediated pathway. Ultrastructural analysis via bio‐EM revealed that SA^p^‐CD28 treated cells displayed nuclear envelope disintegration and chromatin hypercondensation (Figure 3C), while control cells maintained intact nuclear architecture.

The difference in nuclear effects between SA^p^‐CD28 and SA‐CD28^p^ arises from the rate of ALP‐catalyzed dephosphorylation, which leads to distinct structural transformations: As shown in Figure 2B, the dephosphorylation of SA^p^‐CD28 is significantly slower than that of SA‐CD28^p^. Time‐course TEM (Figure S17) corroborates this kinetic difference: upon ALP treatment, SA^p^‐CD28 produces relatively few and short fibrils throughout the reaction window, whereas SA‐CD28^p^ rapidly generates dense, long fibrillar networks. Therefore, it can be inferred that slower dephosphorylation of SA^p^‐CD28 favors the formation of small nanooligomers that can engage CD28 and be internalized [42, 43], while faster dephosphorylation of SA‐CD28^p^ drives rapid fibrillogenesis on the cell surface, producing larger, surface‐bound assemblies that impede internalization. In addition, the acquisition of β‐turn structures in SA^p^‐CD28 is expected to increase molecular rigidity and enhance structural stability [44, 45], which may facilitate cellular uptake of peptides and peptide‐based nanomaterials [39, 46, 47]. This structural feature could also improve the spatial orientation of key residues required for efficient CD28 binding and downstream signaling activation.

The cytotoxicity of SA^p^‐CD28 against Jurkat cells was quantitatively assessed using MTT assays (3‐(4,5‐dimethylthiazol‐2‐yl)‐2,5‐diphenyltetrazolium bromide assay) across a concentration gradient (Figure 3D). SA^p^‐CD28 demonstrated superior cytotoxicity compared to other compounds, reducing cell viability to 20% at 16 µM, in stark contrast to 60% viability observed with SA‐CD28^p^ at the same concentration. SA^p^ and CD28^p^ showed negligible toxicity toward Jurkat cells. To assess biocompatibility with normal cells, human primary CD3^+^ T cells and human hepatocyte cell line L‐02 (LO2) were treated with the four peptides. Notably, all compounds maintained >85% cell viability with no detectable cytotoxicity (Figure 3E; Figure S18), confirming their selective toxicity toward malignant cells. Mechanistic analysis using Annexin V/7‐AAD dual staining revealed necrosis as the predominant cell death pathway (Figure 3F,G). SA^p^‐CD28 induced 78.03% necrosis, compared to 47.87% for SA‐CD28^p^ after 12 h. In contrast, the control peptides SA^p^ and CD28^p^ induced only 20.2% and 23.7% necrosis, respectively. The possible mechanism of cell necrosis is that SA^p^‐CD28 formed oligomer can be endocytosed into the lysosome, and after escaping the lysosome, it enters the cell nucleus. During this process, it is highly likely to further grow into larger aggregates through elongation and secondary nucleation, which physically disrupt the nuclear membrane, leading to cell death [42, 48, 49].

### 2.3 Transcriptomic Profiling Uncovers Mechanisms of Necrotic Cell Death

**FIGURE 4:**
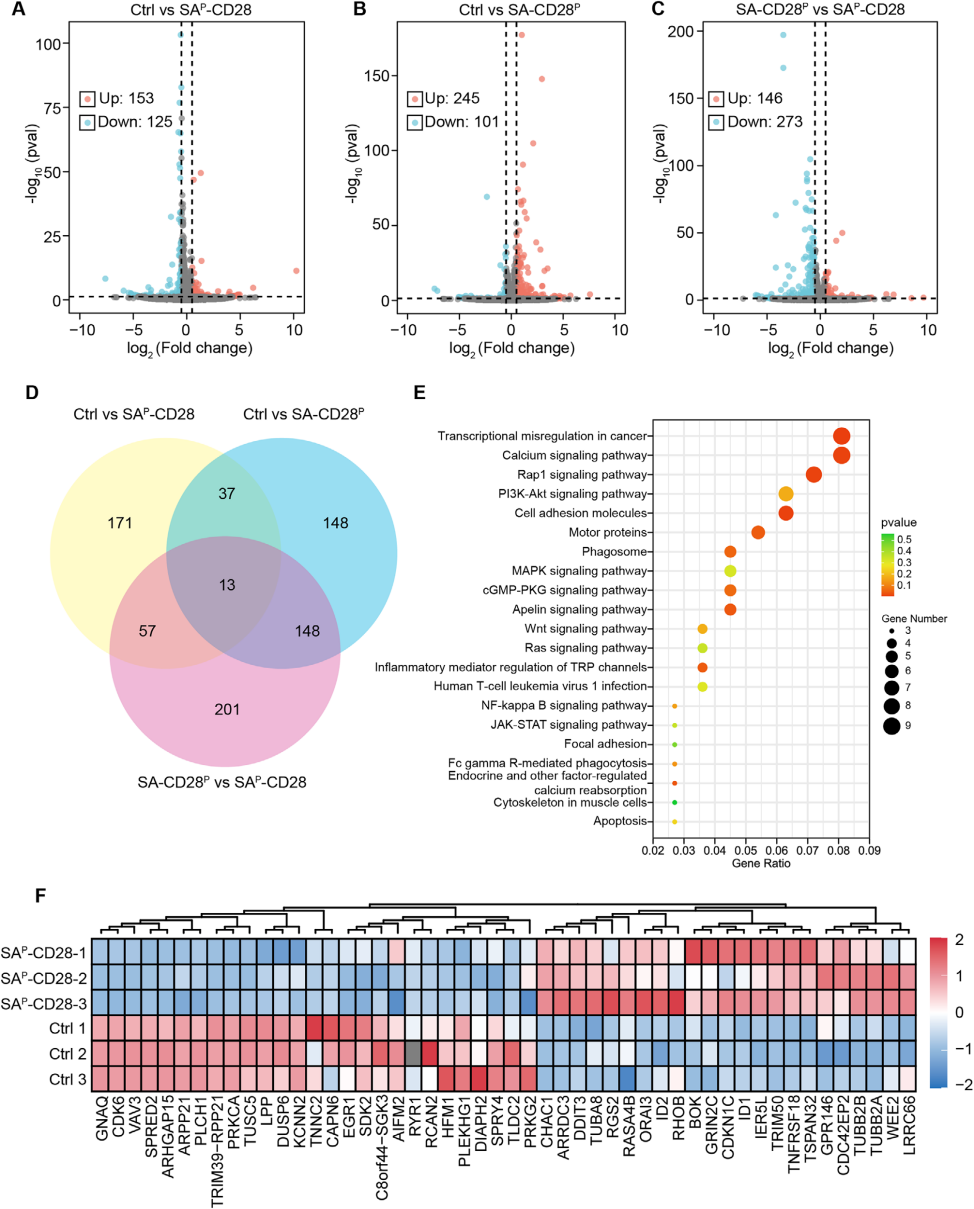
Transcriptomic profiling of cell apoptosis induced by peptides. (A–C) Volcano plot reveals gene regulation in Jurkat cells: Control vs SA^p^‐CD28 (A), Control vs SA^p^‐CD28 (B), and SA‐CD28^p^ vs SA^p^‐CD28 (C). (D) Venn diagram illustrating common and unique deregulated genes in Jurkat cells treated by SA^p^‐CD28 and SA‐CD28^p^ compared with PBS control. (E) KEGG pathway enrichment analysis of the differential pathways in Jurkat cells treated with SA^p^‐CD28 versus PBS Control. (F) The heatmap of typical genes associated with apoptosis and nuclear damage in Jurkat cells after SA^p^‐CD28 treatment in comparison with PBS control.

To further investigate the mechanisms underlying cell necrosis, mRNA transcriptomic profiling was performed using three independent biological replicates per group. The overall sequencing data quality and sample reproducibility were confirmed by RNA integrity assessment and principal component analysis (Figures S19 and S20). Volcano plot analysis (Figure 4A–C) revealed 278 differentially expressed genes (DEGs) in SA^p^‐CD28 treated cells, including 153 upregulated and 125 downregulated genes. In cells treated with SA‐CD28^p^, 346 DEGs were identified, with 245 upregulated and 101 downregulated. Comparative transcriptomic profiling against SA‐CD28^p^ identified 419 DEGs, of which 146 were upregulated, and 273 were downregulated in SA^p^‐CD28 treated cells. Venn diagram analysis (Figure 4D) further demonstrated limited overlap between treatments, with only 50 shared DEGs, while 228 and 296 DEGs were uniquely associated with SA^p^‐CD28 and SA‐CD28^p^, respectively. This marked divergence underscores the distinct bioactivity profile of SA^p^‐CD28.

Kyoto Encyclopedia of Genes and Genomes (KEGG) pathway enrichment analysis (Figure 4E; Figure S21) revealed distinct transcriptomic responses to peptide treatment. Notably, SA^p^‐CD28‐induced nuclear fragmentation appeared to be associated with multiple cellular perturbations, including alterations in calcium signaling, cytoskeletal organization, and inflammatory pathways. Enrichment of calcium signaling pathways (e.g., TRP channel activation) and cytoskeletal remodeling pathways (e.g., focal adhesion) suggested that nuclear envelope instability may arise from broader intracellular signaling disturbances rather than direct nuclear targeting. Meanwhile, pathways related to cell survival signaling, such as the PI3K‐Akt pathway, were also affected, indicating a potential disruption of regulatory networks that coordinate cellular homeostasis and stress responses.

To further evaluate pathway‐level transcriptional trends, Gene Set Enrichment Analysis (GSEA) was performed using the ranked transcriptomic dataset (Figure S22). Notable transcriptional remodeling was observed in several key pathways, including PI3K‐Akt signaling (|NES| = 1.21, *p* = 0.06), Calcium signaling (|NES| = 1.0, *p* = 0.4), and Apoptosis (|NES| = 1.04, *p* = 0.38). Concurrently, NF‐κB signaling and Cell Adhesion Molecules (CAMs) also showed distinct enrichment patterns (|NES| = 1.17 and 1.20, respectively). These findings, although not reaching strict statistical significance (*p* > 0.05), collectively point toward a coordinated shift in the regulatory networks governing cellular survival and structural integrity.

Targeted analysis of 50 pathway‐correlated genes (Figure 4F) provided transcriptional evidence supporting a dual mechanism of SA^p^‐CD28 cytotoxicity: (i) coordinated dysregulation of calcium homeostasis effectors (KCNN2, PLCH1) and cytoskeletal destabilizers (TUBB2A, DIAPH2), contributing to nuclear destabilization. (ii) Suppression of key pro‐survival regulators (e.g., CDK6) coupled with pathological activation of apoptosis‐necrosis transition factors (e.g., BOK), reinforcing the irreversibility of necrosis‐dominant cell death. For comparison, the transcriptional profile of the same 50 pathway‐correlated genes in SA‐CD28^p^‐treated cells was provided in Figure S23.

### 2.4 Validation of Downstream Signaling via CD28 Targeting by SA^p^‐CD28

**FIGURE 5:**
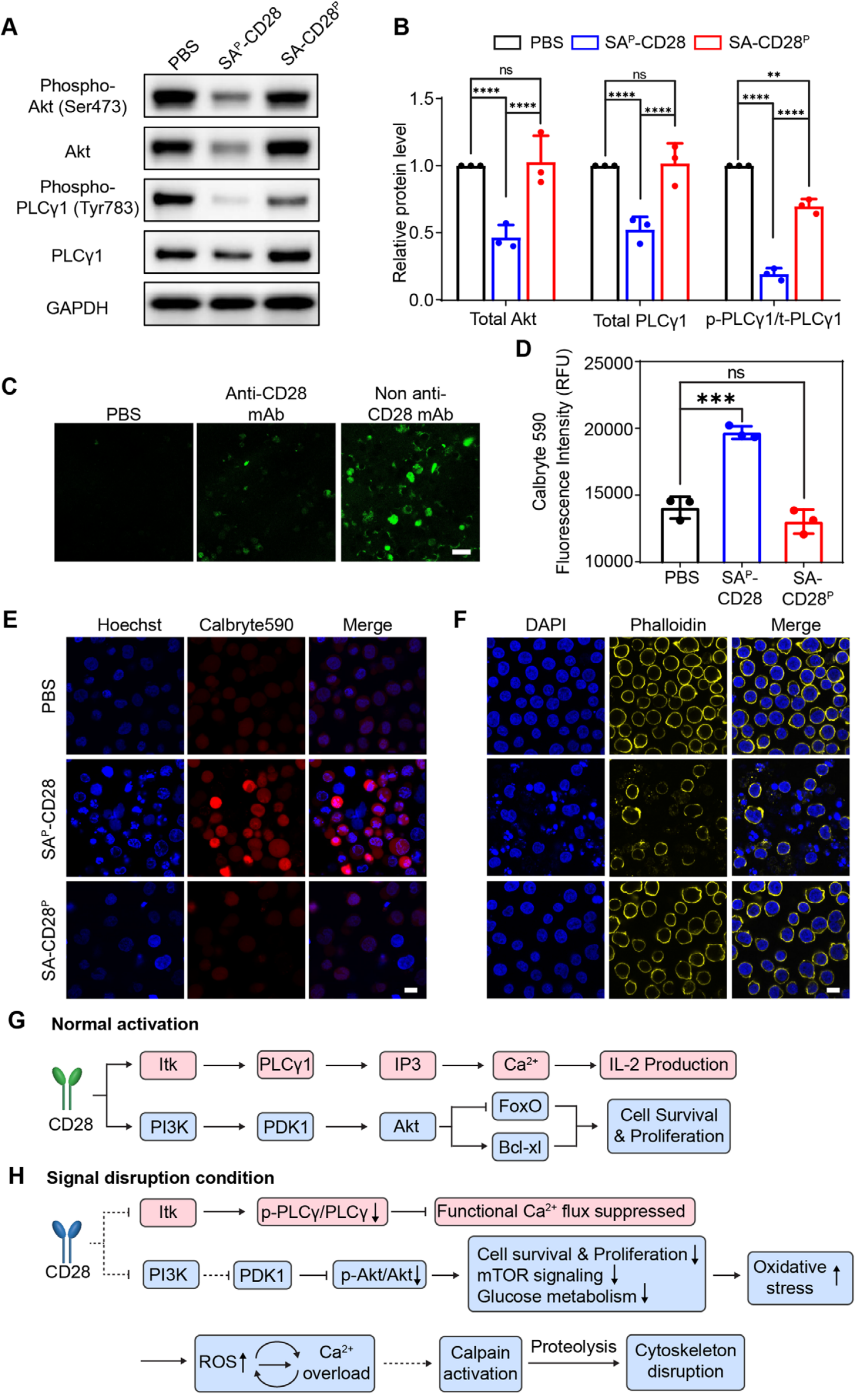
SA^p^‐CD28 disrupts CD28 downstream signaling and induces calcium overload and cytoskeletal collapse. (A, B) Representative Western Blot bands (A) and quantitative analysis (B) of phospho‐Akt, Akt, phospho‐PLCγ, and PLCγ in Jurkat cells treated with SA^p^‐CD28 or SA‐CD28^p^ versus PBS controls. (C) CLSM imaging of Jurkat cells pretreated with anti‐CD28 monoclonal antibody followed by SA^p^‐CD28 treatment. Scale bar: 25 µm. (D, E) Fluorescence microplate quantification (D) and CLSM images (E) of intracellular Ca^2+^ using Calbryte 590 AM after different treatments for 12 h. The red color represented intracellular Ca^2+^, and the blue color indicated the cell nucleus. Scale bar: 10 µm. (F) CLSM imaging of phalloidin‐stained cytoskeleton in cells after 12 h of treatment. The yellow color represented F‐actin, and the blue color indicated the cell nucleus. Scale bar: 10 µm. (G) Canonical CD28 signaling pathway during physiological activation. (H) Proposed mechanism of SA^p^‐CD28‐induced signaling disruption, characterized by suppression of PLCγ and Akt signaling, oxidative stress‐driven Ca^2+^ overload, calpain activation, cytoskeletal disruption, and necrotic cell death. Data are presented as mean ± SD (*n* = 3 independent experiments). Statistical analysis was performed using one‐way ANOVA as ^***^ *p* < 0.001 and ^****^ *p* < 0.0001.

Building upon the transcriptomic findings, which highlighted widespread alterations in calcium signaling, cytoskeletal organization, and survival‐related pathways, two representative downstream signaling axes, PLCγ and PI3K‐Akt, were selected for validation by Western blot analysis. This selection was based on their mechanistic relevance to the observed cellular events. Specifically, KEGG enrichment of calcium signaling and cytoskeletal remodeling pathways suggested that the pronounced nuclear instability might arise from Ca^2+^‐dependent mechanochemical perturbations [50, 51]. As PLCγ lies downstream of CD28 and mediates Ca^2+^ release through PIP_2_ hydrolysis, its activation was examined to determine whether CD28‐PLCγ signaling contributes to the observed calcium dysregulation. PLCγ and PI3K‐Akt are the canonical downstream effectors of CD28 signaling, regulating calcium mobilization and cell survival, respectively. Therefore, their activation states were examined to determine whether disruption of these pathways contributes to the cellular phenotypes predicted by transcriptomic analysis. Meanwhile, the transcriptomic suppression of compensatory survival pathways and upregulation of apoptosis‐necrosis transition factors (e.g., BOK) pointed to a collapse of pro‐survival signaling. Given that the PI3K‐Akt pathway is a central survival axis downstream of CD28, its inhibition was hypothesized to contribute to the loss of cellular homeostasis. Western blot analysis revealed two major signaling alterations (Figure 5A,B). (i) Akt pathway: SA^p^‐CD28 induced substantial degradation of total Akt protein (>54%), indicating pronounced attenuation of pro‐survival signaling. (ii) PLCγ pathway: Total PLCγ1 expression was reduced by 48%,, and the phospho‐PLCγ1 (Tyr783) to total PLCγ1 ratio decreased by approximately 80%, suggesting an overall suppression of PLCγ signaling activity.

To verify that these signaling perturbations were initiated via CD28 engagement, we blocked the receptor with a recombinant anti‐human CD28 monoclonal antibody. This intervention markedly reduced SA^p^‐CD28 internalization (Figure 5C), confirming that SA^p^‐CD28 associates with cells via CD28. To further examine receptor dependence, additional control experiments were performed. In HeLa cells, which exhibit relatively low CD28 expression but retain high alkaline phosphatase activity [29], only minimal peptide uptake and negligible cytotoxicity were observed (Figure S24). Moreover, a scrambled peptide in which the CD28‐targeting motif SPMLVAYD was randomized (SA^p^‐CD28‐Scr, Figure S5) failed to induce appreciable membrane binding or cytotoxicity in Jurkat cells (Figure S25). Collectively, these orthogonal controls support that the cellular activity of SA^p^‐CD28 is associated with CD28 engagement rather than being driven solely by ALP‐mediated peptide activation. Because PLCγ activity was markedly suppressed, the elevated intracellular calcium observed in treated cells is unlikely to originate from canonical CD28‐PLCγ‐IP_3_ signaling. Instead, transcriptomic analysis revealed significant enrichment of the PI3K‐Akt signaling pathway in both KEGG and GSEA analyses, indicating substantial perturbation of this canonical survival axis. Consistent with this observation, Western blot analysis demonstrated a pronounced reduction in total Akt protein levels following SA^p^‐CD28 treatment. Given the central role of Akt in regulating downstream metabolic programs, including mTOR signaling and glucose metabolism, its suppression is expected to impair cellular metabolic homeostasis and redox balance, thereby promoting oxidative stress. Consistent with this notion, measurements of intracellular reactive oxygen species (ROS) demonstrated a significant increase following SA^p^‐CD28 treatment (Figure S26). Oxidative stress is well known to disrupt Ca^2+^ homeostasis by impairing mitochondrial function and endoplasmic reticulum calcium buffering capacity, ultimately leading to pathological Ca^2+^ overload.

Intracellular calcium dynamics were therefore evaluated in Jurkat cells. Calcium imaging was performed using Calbryte 590 AM, a cell‐permeable fluorescent probe that enables visualization of intracellular Ca^2+^ fluctuations in live cells. The results (Figure 5D,E) showed a 1.4‐fold increase in intracellular Ca^2+^ flux (*p* < 0.001) following SA^p^‐CD28 treatment, confirming pathological calcium overload. Notably, ROS accumulation and Ca^2+^ dysregulation are known to reinforce each other through a positive feedback loop, further amplifying cellular stress. Such calcium dysregulation is known to activate calpain, a calcium‐dependent protease that disrupts cytoskeletal integrity [52, 53, 54]. The cytoskeletal integrity of Jurkat cells treated with SA^p^‐CD28 was subsequently examined. The actin cytoskeleton, a fundamental element for cellular integrity, was examined by confocal microscopy following phalloidin staining, which specifically labels filamentous actin (F‐actin). The results revealed pronounced cytoskeletal disintegration, characterized by reduced filament density and fragmented actin networks (Figure 5F). These morphological changes provide visual evidence of calpain‐mediated structural collapse.

To conceptually summarize these signaling alterations, a mechanistic model comparing physiological CD28 signaling with the disrupted signaling state induced by SA^p^‐CD28 is presented in Figure 5G,H. Under normal activation conditions (Figure 5G), CD28 signaling promotes T‐cell survival and proliferation through coordinated activation of the PLCγ‐IP_3_‐Ca^2+^ axis [55]. and the PI3K‐PDK1‐Akt pathway [56, 57], leading to IL‐2 production and maintenance of metabolic homeostasis. In contrast, SA^p^‐CD28 induces a distinct signaling state characterized by suppression of both PLCγ and Akt pathways (Figure 5H). Inhibition of Akt signaling compromises cellular metabolism and mTOR activity, leading to oxidative stress [58]. The resulting ROS accumulation disrupts intracellular Ca^2+^ homeostasis, producing Ca^2+^ overload that activates calpain and triggers proteolytic cytoskeletal breakdown. Together, these events culminate in nuclear fragmentation and necrotic cell death.

Collectively, these findings establish a sequential mechanism in which SA^p^‐CD28 engagement of CD28 disrupts canonical survival signaling, leading to metabolic collapse, oxidative stress, Ca^2+^ overload, and calpain‐mediated cytoskeletal destruction, ultimately driving necrotic cell death.

### 2.5 Evaluation of Antitumor Activity in BALB/c Nude Mice With Jurkat T‐ALL Xenograft Tumors

**FIGURE 6:**
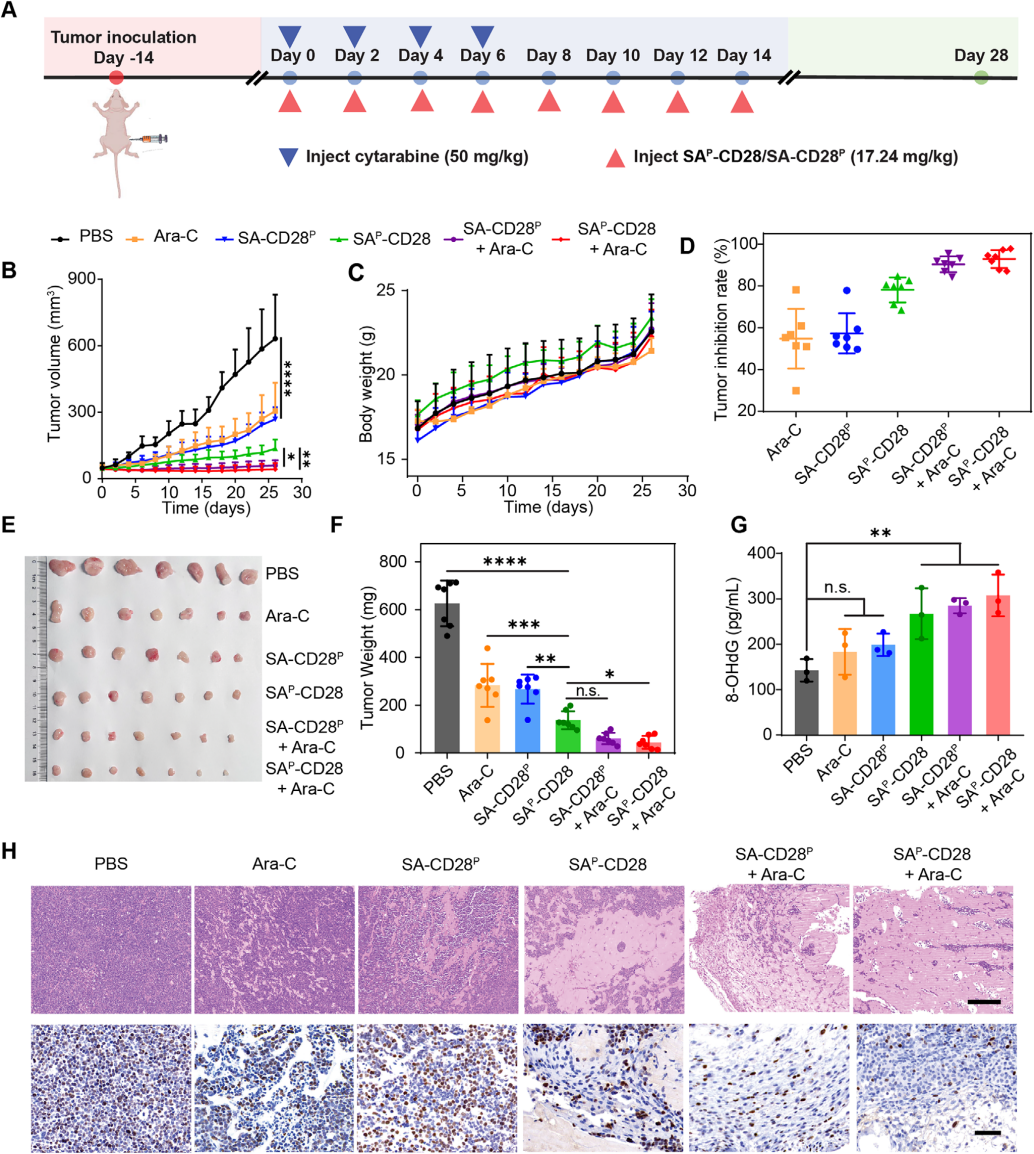
Antitumor effects of the self‐assembling peptides in Jurkat tumor‐bearing nude mice. (A) Schematic illustration of tumor inoculation and treatment in mice. (B) The growth curves of the tumors during the treatments. (C) The variations in mouse weights during the indicated treatments. (D) Tumor inhibition rates are determined by measuring tumor volumes at the end of treatment. (E) The digital images of the dissected tumors after the indicated treatments. (F) The average tumor weight at the end of the treatment. (G) Quantification of serum 8‐OHdG as an oxidative stress biomarker in mice at the therapeutic endpoint. (H) Representative hematoxylin and eosin (H&E, top panel; scale bar: 200 µm) and Ki‐67 immunohistochemistry (Ki‐67, bottom panel; scale bar: 50 µm) staining of tumor tissues at the study endpoint after the indicated treatments. Data are presented as mean ± SD (*n* = 7 mice per group for B‐D, F; *n* = 3 mice per group for (G)). Statistical significance was determined using two‐way ANOVA (B) or one‐way ANOVA (F, G). ^*^ *p* < 0.05, ^**^ *p* < 0.01, ^***^ *p* < 0.001, ^****^ *p* < 0.0001.

To evaluate the in vivo antitumor efficacy of SA^p^‐CD28, Jurkat xenografts were first established in BALB/c nude mice. The treatment regimen is shown in Figure 6A. After tumors reached 50 mm^3^, mice (*n* = 7 per group) were randomized into different treatment groups. SA^p^‐CD28 was administered intravenously at 17.24 mg/kg every two days (q2d) for a total of eight doses to assess its monotherapeutic activity. To further potentiate tumor suppression, cytarabine (50 mg/kg, intraperitoneal injection, q2d × 4) was co‐administered according to the indicated schedule. The dosing regimen was selected based on previously reported protocols in murine leukemia models [59, 60]. Control groups received saline (negative control), cytarabine (Ara‐C) alone (50 mg/kg, i.p., q2d × 4), or SA‐CD28^p^ alone at an equivalent dose. Tumor volume and body weight were monitored across the 28‐day treatment period.

As shown in Figure 6B, D–F. SA^p^‐CD28 monotherapy achieved a tumor growth inhibition rate of 78.09% (137.24 mg), outperforming SA‐CD28^p^ by 36.5% (*p* = 0.0003), which produced only 57.28% inhibition (255.02 mg). Cytarabine alone resulted in 54.76% inhibition (306.9 mg). Importantly, the sequential combination of SA^p^‐CD28 and cytarabine produced a synergistic effect, reaching 92.9% tumor inhibition (34.19 mg) and inducing measurable tumor regression (from 45.07 to 43.44 mm^3^), a therapeutic outcome unattainable with either agent alone. Meanwhile, body weight (Figure 6C) remained stable in all treatment groups (*p* > 0.05), indicating favorable safety.

In vitro studies demonstrated that SA^p^‐CD28 elicited both oxidative and calcium‐mediated stress responses. To determine whether this mechanism also occurred in vivo, we measured serum 8‐OHdG, a biomarker of DNA oxidative damage [61]. The results showed an approximately two‐fold increase in 8‐OHdG levels (Figure 6G) in the SA^p^‐CD28 and combination groups (*p* < 0.01 vs PBS), suggesting oxidative stress contributes to tumoricidal activity. Hematoxylin and eosin (H&E) staining (Figure 6H) revealed extensive necrosis in SA^p^‐CD28‐treated tumors, with necrotic regions exceeding 50% in the combination group. Correspondingly, Ki67 expression (Figure 6H) dropped to 20% in the combination treatment compared to >80% in PBS controls, demonstrating near‐complete proliferative arrest. H&E staining of major organs (heart, liver, spleen, lung, and kidney) at the study endpoint showed no observable histopathological abnormalities (Figure S27), further supporting the biocompatibility of the tested peptides and materials.

Collectively, these results indicate that SA^p^‐CD28 exerts potent nanotherapeutic effects against T‐ALL xenografts by inducing coordinated mechanical and oxidative stress. The addition of cytarabine further amplifies this effect by simultaneously targeting biochemical (DNA synthesis) and biophysical (cytoskeletal‐nuclear integrity) vulnerabilities, forming a dual‐targeting strategy that overcomes compensatory resistance mechanisms.

## 3 Conclusion

This study identifies a new target, CD28, for T‐ALL therapy. A modular enzyme‐responsive self‐assembling peptide was designed to selectively bind to CD28, intervene in the Akt and PLCγ signaling pathways, and utilize self‐assembled peptides to enter the cell nucleus and physically disrupt the nucleus to kill cancer cells. This molecule undergoes an enzyme‐responsive cleavage to release phosphate groups, resulting in a conformational change from α‐helix to β‐sheet/β‐turn‐dominated nanostructures (80.3% β‐sheet, 17.9% β‐turn), which enhances CD28 binding affinity by 32.4‐fold (K_D_ value from 283.7 to 8.748 µM). Moreover, this structural transformation leads to the self‐assembly of nanooligomers on the cell membrane, which, upon cellular entry, further elongates to disrupt the cell nucleus, ultimately causing cancer cell death. In summary, the peptide designed in this study employs three distinct pathways to kill tumors: (1) dysregulation of PLCγ signaling suppresses canonical Ca^2+^ signaling pathways; (2) suppression of the Akt signaling pathway induces oxidative stress and disrupts Ca^2+^ homeostasis, leading to Ca^2+^ overload‐mediated cytoskeletal collapse and necrotic cell death; and (3) physical nuclear disruption via large self‐assembled nanostructures. This morphology‐based molecular design strategy, with its multi‐path tumor‐killing mechanism, holds great therapeutic potential for cancers with few druggable targets in the future.

## Author Contributions

J.L. contributed to writing – original draft, data curation, project administration, methodology, and conceptualization; Z.J. and D.L. contributed to investigation, validation, data curation, and formal analysis and contributed equally to this work; Z.‐W.H. contributed to methodology and conceptualization; Y.D. contributed to methodology and data curation; S.Z. and S.T. contributed to investigation and data curation; Z.Y. contributed to funding acquisition; H.F. contributed to project administration; L.W. contributed to conceptualization, supervision, and project administration; and M.‐D.W. contributed to conceptualization, supervision, writing – original draft, and project administration. All authors have read and approved the final version of the manuscript.

## Ethics Approval and Consent to Participate

All animal experiments were approved by the Animal Experiments Ethics Committee of Nankai University. (accreditation No.: 2022‐SYDWLL‐000418). All animal experiments were conducted in strict accordance with the guidelines for the management and use of experimental animals by this institution.

## Conflicts of Interest

The authors declare no conflicts of interest.

## Supporting information




**Supporting file**: advs75052‐sup‐0001‐SuppMat.docx

## Data Availability

The data that support the findings of this study are available in the supplementary material of this article.
