# Supplementary material for: CD28‐Targeted Enzyme‐Responsive Conformation‐Switching Peptide Self‐Assembly for Selective T‐Cell Acute Lymphoblastic Leukemia (T‐ALL) Therapy
Source: Adv Sci (Weinh). 2026 Apr 2;13(34):e20963. doi: 10.1002/advs.202520963 (PMC13285142; doi:10.1002/advs.202520963)
Supplement: Supplementary file 1 — Supporting file: advs75052‐sup‐0001‐SuppMat.docx [file ADVS-13-e20963-s001.docx]

**Supporting Information**

**CD28-Targeted Enzyme-Responsive Conformation-Switching Peptide Self-Assembly for Selective T-cell acute lymphoblastic leukemia (T-ALL) Therapy**

Jun Li,^[a]^ Ziyu Jia,^[a]^ Dekun Li,^[a]^ Zhi-Wen Hu,^[a]^ Yinghao Ding,^[a]^ Shengyi Zhang,^[a]^ Shengkun Tuo,^[a]^ Zhimou Yang,^[a]^ Huisheng Fu,*^[b]^ Ling Wang,*^[a]^ Man-Di Wang*^[a]^

[a] Jun Li, Ziyu Jia, Dekun Li, Zhi-Wen Hu, Yinghao Ding, Shengyi Zhang, Shengkun Tuo, Zhimou Yang*, Ling Wang*, Man-Di Wang*

State Key Laboratory of Medicinal Chemical Biology, College of Pharmacy, Nankai University, Tianjin 300071, P. R. China

E-mail: chwling@nankai.edu.cn; wangmd@nankai.edu.cn

[b] Huisheng Fu*

Hemodialysis Center, Department of Nephrology, Tianjin Medical University General Hospital, Tianjin 300052

E-mail: shenghui1974@126.com

Table of Contents

[1. Materials and Methods 4](#_Toc224561469)

[**1.1 Animals** 4](#_Toc224561470)

[**1.2 Materials** 4](#_Toc224561471)

[**1.3 Cell lines and cell culture** 5](#_Toc224561472)

[**1.4 Solid-Phase Peptide Synthesis** 5](#_Toc224561473)

[**1.5 Peptide Solution Preparation** 6](#_Toc224561474)

[**1.6 The measurement of the dephosphorylation kinetics** 6](#_Toc224561475)

[**1.7 Transmission Electron Microscopy (TEM)** 7](#_Toc224561476)

[**1.8 Circular Dichroism (CD) Spectroscopy** 7](#_Toc224561477)

[**1.9 Fourier-Transform Infrared Spectroscopy (FTIR)** 7](#_Toc224561478)

[**1.10 Microscale Thermophoresis (MST)** 8](#_Toc224561479)

[**1.11 Confocal Laser Scanning Microscopy (CLSM)** 9](#_Toc224561480)

[**1.12** **Endocytosis Pathway Assay** 9](#_Toc224561481)

[**1.13** **Cytotoxicity Measurements In Vitro** 9](#_Toc224561482)

[**1.14 Biocompatibility Assay** 10](#_Toc224561483)

[**1.15 Flow Cytometric Analysis of Apoptosis** 10](#_Toc224561484)

[**1.16 Transcriptomic Analysis** 10](#_Toc224561485)

[**1.17 Western Blot** 11](#_Toc224561486)

[**1.18 Calcium Signaling Imaging** 12](#_Toc224561487)

[**1.19 Cytoskeleton Staining** 12](#_Toc224561488)

[**1.20 Animal Experiments** 12](#_Toc224561489)

[**1.21 Hematoxylin and Eosin (H&E) Staining** 13](#_Toc224561490)

[**1.22 8-Hydroxy-2'-Deoxyguanosine (8-OHdG) Detection** 13](#_Toc224561491)

[**1.23 Statistical Analysis** 13](#_Toc224561492)

[2. Supplementary Figures 15](#_Toc224561493)

[**2.1 Chemical structure and ESI-MS spectra of peptides** 15](#_Toc224561494)

[**2.2 FTIR and CD Spectroscopic Analysis of Peptide Conformation** 20](#_Toc224561495)

[**2.3 Raw MST traces and thermophoresis time window selection** 23](#_Toc224561496)

[**2.4 MST Binding Curves of Peptides with and without ALP** 24](#_Toc224561497)

[**2.5 Measurement of alkaline phosphatase secretion and CD28 expression levels in human cell lines** 25](#_Toc224561498)

[**2.6 Quantitative Analysis and Imaging of Peptide Uptake in Jurkat Cells** 27](#_Toc224561499)

[**2.7 Lysosomal Colocalization and Escape of SA^p^-CD28** 29](#_Toc224561500)

[**2.8 Quantitative analysis of the endocytosis mechanism of SA^p^-CD28** 30](#_Toc224561501)

[**2.9 Time-dependent TEM characterization of enzymatic assembly** 31](#_Toc224561502)

[**2.10 Cytotoxicity Assessment of Peptides in LO2 Cells** 32](#_Toc224561503)

[**2.11 Quality control of RNA sequencing samples and transcriptomic data** 33](#_Toc224561504)

[**2.12 KEGG Pathway Enrichment and Gene Expression Heatmap of SA-CD28^p^-Treated Jurkat Cells** 37](#_Toc224561505)

[**2.13** **Control experiments supporting the CD28-dependent activity of SA^p^-CD28** 40](#_Toc224561506)

[**2.14 Detection and Quantification of Intracellular ROS Levels** 42](#_Toc224561507)

[**2.15 Histological Examination of Major Organs Across Treatment Groups** 43](#_Toc224561508)

[References 44](#_Toc224561509)

**1. Materials and Methods**

**1.1 Animals**

All animal experiments were approved by the Animal Ethics Committee of Nankai University and conducted in accordance with the guidelines of the Tianjin Laboratory Animal Use and Care Committee. BALB/c nude mice (3 weeks old) were purchased from Beijing Vital River Laboratory Animal Technology Co., Ltd. (Beijing, China). All mice were maintained under specific-pathogen-free (SPF) conditions at the Nankai Animal Resources Center. All procedures were approved by the Animal Ethics Committee of Nankai University.

**1.2 Materials**

Fmoc-protected amino acids and o-benzotriazol-1-yl-N,N,N′,N′-tetramethyluronium hexafluorophosphate (HBTU) were obtained from GL Biochem (Shanghai, China). 2-Chlorotrityl chloride resin (1.1 mmol g^-1^) was purchased from Nankai University Resin Co. (Tianjin, China). N,N-Diisopropylethylamine (DIPEA) was obtained from Energy Chemical (Shanghai, China). NBD chloride (NBD-Cl) was purchased from Tokyo Chemical Industry Co., Ltd. (TCI, Tokyo, Japan). NBD-β-Ala was synthesized according to our previous literature.^[1]^ Alkaline phosphatase (30 U/μL) was purchased from Takara Biomedical Technology (Beijing, China). Dulbecco’s modified Eagle’s medium (DMEM) and penicillin/streptomycin were obtained from Gibco (Grand Island, NY, USA). Recombinant human CD28 protein (RP00136) was purchased from ABclonal (Wuhan, China). The Monolith NT^TM^ Protein Labeling Kit BLUE-NHS was obtained from NanoTemper Technologies (Munich, Germany). The H5N1 Alkaline Phosphatase Assay Kit (P0321S) was purchased from Beyotime Biotechnology (Shanghai, China). FITC-conjugated anti-human CD28 antibody was obtained from BioLegend (San Diego, CA, USA). Thiazolyl Blue (MTT, T51500) was purchased from Tianjin Xiensi Biochemical Technology Co., Ltd. (Tianjin, China). The Annexin V Apoptosis Detection Kit (88-8102-72) was obtained from Thermo Fisher Scientific (Waltham, MA, USA). Antibodies against phospho-PLCγ1 (Tyr783, #2821), PLCγ1 (D9H10, #5690), phospho-Akt (Ser473, D9E, #4060), and Akt (pan, C67E7, #4691) were purchased from Cell Signaling Technology (Danvers, MA, USA). Calbryte™ 590 AM was obtained from Biolite (Xi’an, China). TRITC-phalloidin (40734ES75) was purchased from Yeasen Biotechnology (Shanghai, China). Cytarabine (HY-13605) was obtained from MedChemExpress (Monmouth Junction, NJ, USA).

**1.3 Cell lines and cell culture**

The human T lymphocyte cell line (Jurkat) was purchased from Cyagen Biosciences (Guangzhou) Inc. (Guangzhou, China). The human normal liver cell line (LO2), cervical cancer cell line (HeLa), human proximal tubular epithelial cell line (HK-2), and human umbilical vein endothelial cells (HUVEC) were obtained from our laboratory stocks. Primary human CD3^+^ T cells were broadly kindly provided by Dr. Jian Wen (Wenzhou Medical University, Wenzhou, China).

Jurkat and LO2 cells were cultured in RPMI-1640 medium supplemented with 10% fetal bovine serum (FBS) and 1% penicillin-streptomycin. HeLa, HK-2 and HUVEC cells were cultured in Dulbecco’s Modified Eagle Medium (DMEM) supplemented with 10% FBS and 1% penicillin–streptomycin. All cells were cultured at 37 °C in a humidified incubator with 5% CO₂.

**1.4 Solid-Phase Peptide Synthesis**

Target peptides were synthesized by standard Fmoc-based solid-phase peptide synthesis (SPPS) on 2-chlorotrityl chloride resin (substitution: 1.1 mmol g^-1^). Conventional Fmoc-protected amino acids and Fmoc-Tyr (PO_3_Bzl) were used to incorporate the normal and phosphorylated residues, respectively. The Fmoc protecting groups were removed with 20% piperidine in N,N-dimethylformamide (DMF), and O-benzotriazol-1-yl-N,N,N′,N′-tetramethyluronium hexafluorophosphate (HBTU) was employed as the coupling reagent. After iterative deprotection and coupling cycles, the crude peptides were cleaved from the resin using a mixture of trifluoroacetic acid (TFA)/triisopropylsilane (TIS)/H_2_O (95:2.5:2.5, v/v/v), precipitated in cold diethyl ether, and purified by reversed-phase high-performance liquid chromatography (RP-HPLC, Agilent ZORBAX SB-C18, 5 μm, 9.4 × 250 mm) using a linear gradient of methanol/water containing 0.1% TFA. The purified peptides (>95% purity) were characterized by LC-MS (LCMS-2020, Shimadzu, Japan) and liquid chromatography-quadrupole time-of-flight mass spectrometry (LC-QTOF-MS, Agilent, USA). The final products were obtained by lyophilization.

**1.5 Peptide Solution Preparation**

Lyophilized peptides (1 mg) were dissolved in phosphate-buffered saline (PBS, pH 7.4) to a final concentration of 1 mM in 2 mL glass vials. The pH was fine-tuned to 7.4 using 2 M sodium carbonate (Na_2_CO_3_). Peptide solubility was evaluated visually and documented through photography. Subsequently, alkaline phosphatase (ALP, 5 U mL^-1^) was added, and the samples were incubated at 37 °C for 12 h. The Tyndall effect was examined under laser irradiation, and optical images were recorded.

**1.6 The measurement of the dephosphorylation kinetics**

A 1 mM peptide stock solution in deionized water was preincubated at 37 °C for 30 min. Alkaline phosphatase (ALP, 2 U mL⁻¹, final concentration) was then added to initiate the reaction. Aliquots (20 μL) were withdrawn at predetermined time points, mixed with 180 μL methanol to quench the reaction, and further diluted prior to LC-MS (LCMS-2020, Shimadzu, Japan) analysis. Dephosphorylation rates were quantified by LC-MS based on the relative peak intensities of phosphorylated and dephosphorylated species. The dephosphorylation rate was quantified by manual integration of the peaks corresponding to the phosphorylated and dephosphorylated species at an absorption wavelength of 220 nm. Data analysis was performed using GraphPad Prism 8.0.

**1.7 Transmission Electron Microscopy (TEM)**

Microscopic morphologies of peptide assemblies were examined by transmission electron microscopy (TEM). Typically, 10 μL of sample solution was dropped onto a carbon-coated copper grid and allowed to adsorb for 1 min at room temperature. The excess solution was blotted with filter paper, and the grid was negatively stained with 2% (w/v) uranyl acetate for 1 min. The grid was then blotted again to remove excess stain and air-dried in a desiccator overnight. TEM images were acquired on a Talos L120C G2 microscope operated at 120 kV, and the diameters of the peptide nanofibers were quantified using ImageJ software.

**1.8 Circular Dichroism (CD) Spectroscopy**

Secondary structural changes of the peptides, with or without ALP treatment, were analyzed using a MOS-450 circular dichroism (CD) spectrometer (BioLogic). Peptide stock solution (1 mM, deionized water) was diluted to 50 μM for CD measurement. Spectra were recorded from 190 to 260 nm in a 0.3 mm quartz cuvette (step size: 1 nm; integration time: 1 s) at 25 °C. Raw data were processed with GraphPad Prism 8.0, and secondary structure contents were estimated using the DichroWeb online analysis server (http://dichroweb.cryst.bbk.ac.uk). Structural composition ratios (α-helix, β-sheet, β-turn, random coil) were determined via the standard SELCON3 algorithm.

**1.9 Fourier-Transform Infrared Spectroscopy (FTIR)**

Freeze-dried peptide samples with or without ALP treatment were mixed with potassium bromide (KBr) at a mass ratio of 1:100 (w/w) and pressed into transparent pellets. Fourier-transform infrared (FTIR) spectra were recorded on a TENSOR 37 spectrometer (Bruker, Germany) over the range of 400-4000 cm^-1^. Secondary structural features were analyzed based on the amide I band (1600-1700 cm^-1^).

**1.10 Microscale Thermophoresis (MST)**

Recombinant human CD28 protein was fluorescently labeled using a Monolith NT.115 Protein Labeling Kit BLUE-NHS (Amine Reactive) according to the instructions of manufacturer. Briefly, the dye containing an NHS-ester reactive group was conjugated to the primary amines of the protein. A 4 mM peptide solution in PBS was serially twofold diluted to obtain 16 concentrations. Labeled human CD28 protein was then mixed with each peptide dilution at a 1:1 volume ratio, and the mixtures were then aspirated with capillaries for the subsequent measurements. Microscale thermophoresis measurements were performed using a Monolith NT.115 instrument (NanoTemper Technologies, Germany). MST data were analyzed using NanoTemper Analysis software. For each experimental set, the thermophoresis time window was selected within the stable thermophoretic phase and was kept constant across all ligand concentrations to ensure consistency in signal extraction. For each MST experiment, the thermophoresis time window was selected within the stable thermophoretic phase and was kept identical for all ligand concentrations within the same experimental set to ensure consistent data analysis. Binding affinities were determined using the built-in K_D_ fitting model. As ALP treatment induces peptide dephosphorylation and promotes multimeric assembly, the resulting K_D_ values are reported as apparent dissociation constants reflecting the overall interaction strength between peptide assemblies and CD28 rather than a strictly monomeric binding interaction.

**1.11 Confocal Laser Scanning Microscopy (CLSM)**

Jurkat cells (8 × 10^5^ cells per dish) were seeded in poly-L-lysine-coated confocal dishes and incubated overnight. ^[2-4]^The cells were then treated with fresh medium containing the indicated peptides (100 µM) for 12 h. Following treatment, the cells were washed three times with phosphate-buffered saline (PBS) and stained with Hoechst 33342 (C1025, Beyotime) for 30 min in the dark. After additional PBS washes, fluorescence images were acquired using a TSC SP8 (Leica, Germany) confocal microscope.

**1.12** **Endocytosis Pathway Assay**

Jurkat cells were seeded in six-well plates at a density of 5 × 10^5^ cells per well and allowed to adhere overnight. On the following day, the cells were pretreated with endocytosis inhibitors, including Nocodazole (3.3 μM), M-β-cyclodextrin (M-β-CD, 28.2 μM), Filipin III (2 μM), 5-(N-ethyl-N-isopropyl)-amiloride (EIPA, 20 μM), and Dynasore (40 μM), in a humidified incubator at 37 °C with 5% CO_2_ for the specified duration. Subsequently, the cells were incubated with SA^p^-CD28 (100 μM) for 1 h. After two washes with phosphate-buffered saline (PBS), the fluorescence intensity of Jurkat cells was measured by flow cytometry.

**1.13** **Cytotoxicity Measurements In Vitro**

Jurkat cells were seeded in 96-well plates at a density of 8 × 10^4^ cells per well and incubated at 37 °C with 5% CO_2_ for 12 h. The culture medium was then replaced with RPMI-1640 containing the peptides at concentrations ranging from 2 to 16 μM. Following a 48-h incubation, the medium was replaced with RPMI-1640 supplemented with 0.5 mg/mL MTT, and the cells were incubated for an additional 4 h. The medium was subsequently removed, and 100 μL of dimethyl sulfoxide (DMSO) was added to each well. Absorbance was measured at 570 nm using a microplate reader (Bio-Rad iMark, USA), and the results were analyzed with GraphPad Prism 8.0.

**1.14 Biocompatibility Assay**

Normal human CD3^+^ T cells were seeded in 96-well plates at a density of 1 × 10^6^ cells per well and incubated overnight. The culture medium was then replaced with RPMI-1640 containing the peptides at concentrations of 2-16 μM, and the cells were incubated for 48 h. Subsequently, the medium was replaced with RPMI-1640 supplemented with 0.5 mg/mL MTT, and the cells were incubated for an additional 4 h. The medium was removed, and 100 μL of dimethyl sulfoxide (DMSO) was added to each well. Absorbance at 570 nm was measured using a microplate reader (Bio-Rad iMark, USA), and the data were analyzed using GraphPad Prism 8.0.

**1.15 Flow Cytometric Analysis of Apoptosis**

Jurkat cells were seeded in six-well plates at a density of 5 × 10^5^ cells per well and incubated overnight. The cells were then treated with the indicated peptides (100 μM) for 12 h. After treatment, the cells were collected, washed with phosphate-buffered saline (PBS), and stained with an Annexin V apoptosis detection kit (Thermo Fisher Scientific) in the dark at 4 °C according to the manufacturer’s instructions. Necrotic and apoptotic populations were analyzed by flow cytometry (LSR Fortessa, BD Biosciences, USA), and the data were processed using GraphPad Prism 8.0.

**1.16 Transcriptomic Analysis**

Jurkat cells (5 × 10^6^ cells per T25 flask) were cultured under standard conditions and treated with PBS (vehicle control) or the indicated peptides (SA^p^-CD28 and SA-CD28^p^, 100 μM each) for 5 h. Total RNA was extracted using TRIzol reagent. RNA integrity was assessed by agarose gel electrophoresis, and all samples exhibited intact 28S and 18S rRNA bands without detectable degradation (Figure S18). RNA libraries were prepared and sequenced by Shanghai Weihuan Biotechnology Co., Ltd. Raw sequencing reads were filtered to remove adaptor sequences and low-quality reads prior to downstream analysis. The sequencing data exhibited high quality, with Q30 percentages exceeding 94% (Table S1), overall mapping rates above 97%, and unique mapping rates above 95%. Clean reads were aligned to the reference human genome, and gene expression levels were quantified for differential expression analysis. Differentially expressed genes (DEGs) were identified using the criteria |log_2_FC| ≥ 1 with Benjamini-Hochberg false discovery rate (FDR) correction, and genes with adjusted p values < 0.05 were considered significantly differentially expressed. Principal component analysis (PCA) was performed to evaluate the reproducibility among biological replicates (Figure S19). Functional enrichment analysis, including KEGG pathway enrichment and gene set enrichment analysis (GSEA), was performed using the online analysis platform (https://cloud.apexbio.cn/). The raw RNA-seq data have been deposited in the Gene Expression Omnibus (GEO) database under accession number GSE322735.

**1.17 Western Blot**

Jurkat cells were seeded into 6-well plates (8 × 10^5^ cells per well) and treated with 100 μM peptide solutions for 8 h. Cells were centrifuged (1200 rpm, 5 min, 4 °C) and washed once with cold PBS. Whole-cell lysates were prepared with RIPA buffer (P0013B, Beyotime) supplemented with a protease inhibitor cocktail (P1045, Beyotime). Samples were lysed on ice for 30-60 s, then centrifuged at 12,000 rpm for 15 min at 4 °C. Protein concentrations were determined using a BCA kit (E112-02, Vazyme). Equal amounts (30 μg) of protein were loaded for SDS-PAGE and transferred for Western blotting. Primary antibodies were used at the following dilutions: Akt (1:1000, CST), Phospho-Akt (Ser473, 1:1000, CST), PLCγ1 (1:1000, CST), Phospho-PLCγ1 (Tyr783, 1:1000, CST), and GAPDH (1:5000). HRP-conjugated secondary antibodies (Abs20039 and Abs20040, Absin) were used at 1:2000. Bands were visualized and quantified using ImageJ.

**1.18 Calcium Signaling Imaging**

Jurkat cells (8 × 10^5^ cells per dish) were seeded in poly-L-lysine-coated confocal dishes and incubated with Calbryte^TM^ 590 AM dye (1×) for 60 min at 37 °C. After washing with HHBS, the cells were treated with peptide solutions (100 μM) for 12 h. Intracellular calcium flux was visualized using a confocal laser scanning microscope (TCS SP8, Leica, Germany) and quantified at an excitation/emission wavelength of 540/590 nm using a fluorescence microplate reader (Spark, Tecan, Austria).

**1.19 Cytoskeleton Staining**

Cells were harvested and washed three times with PBS, then fixed with 4% paraformaldehyde at room temperature for 20 min. After fixation, cells were washed three times with PBS. Permeabilization was performed with 0.4% Triton X-100 in PBS for 10 min, followed by three PBS washes. F-actin was stained with TRITC-phalloidin (0734ES75, Yeasen) according to previously described protocols. ^[5, 6]^After washing 2-3 times with PBS, samples were mounted with antifade medium containing DAPI for nuclear staining and imaged by confocal microscopy (TCS SP8, Leica, Germany).

**1.20 Animal Experiments**

Male BALB/c nude mice (3 weeks old) were acclimated for one week prior to experimentation. Jurkat cells in the logarithmic growth phase were washed with RPMI-1640, resuspended in Matrigel,^[7, 8]^ and kept on ice. A suspension containing 1 × 10^7^ cells in 100 μL was subcutaneously injected into the right flank of each mouse. When tumor volumes reached approximately 50 mm^3^ (≈14 days post-inoculation), mice were randomly assigned to different treatment groups, with Day 0 defined as the start of treatment. Tumor volumes were calculated according to the formula V = (length × width^2^)/4. After 28 days of treatment, mice were anesthetized and euthanized. Tumors and major organs were excised, weighed, photographed, and fixed in 10% neutral-buffered formalin at 4 °C for 24 h prior to further analysis. The primary experimental endpoints, including tumor volume and overall survival, were predefined prior to study initiation. Tumor volume was monitored longitudinally, and survival was recorded according to humane endpoint criteria approved by the institutional animal care guidelines.

**1.21 Hematoxylin and Eosin (H&E) Staining**

Tumor and organ tissues were paraffin-embedded, sectioned, and deparaffinized in xylene for 10 min. Sections were immersed sequentially in graded ethanol solutions (100%, 95%, 90%, 80%, 70%, 5 min each) and rinsed with distilled water. Nuclei were stained with hematoxylin for 5 min, blued in saline acetate, and rinsed with tap water. Cytoplasm was counterstained with eosin for 3 min. Sections were dehydrated, mounted, and imaged by light microscopy.

**1.22 8-Hydroxy-2'-Deoxyguanosine (8-OHdG) Detection**

Blood samples were collected from the orbital sinuses of mice and allowed to clot at room temperature for 2 h. Serum was obtained by centrifugation (3000 rpm, 15 min, 4 °C), and 8-hydroxy-2'-deoxyguanosine (8-OHdG) levels were measured using a commercial ELISA kit to assess oxidative stress in vivo.

**1.23 Statistical Analysis**

All statistical analyses were performed using GraphPad Prism (version 8.0) or Origin (version 2022). Data distribution was assessed for normality using the Shapiro-Wilk test. For normally distributed data, comparisons between two groups were conducted using unpaired two-tailed Student’s t-test, with Welch’s correction applied when variances were unequal. Comparisons among multiple groups were performed using one-way or two-way analysis of variance (ANOVA) followed by appropriate post hoc multiple-comparison tests (Tukey’s or Bonferroni’s test). For data that did not meet normality assumptions, non-parametric tests were applied, including the Mann-Whitney U test for two-group comparisons and the Kruskal-Wallis test followed by Dunn’s multiple-comparison test for multiple groups. Data are presented as mean ± standard deviation (mean ± SD) unless otherwise specified. Exact p values are provided in the figure legends or main text where appropriate. Statistical significance was defined as follows: *p < 0.05, **p < 0.01, ***p < 0.001; ****p < 0.0001; ns, not significant.

**2. Supplementary Figures**

**2.1 Chemical structure and ESI-MS spectra of peptides**


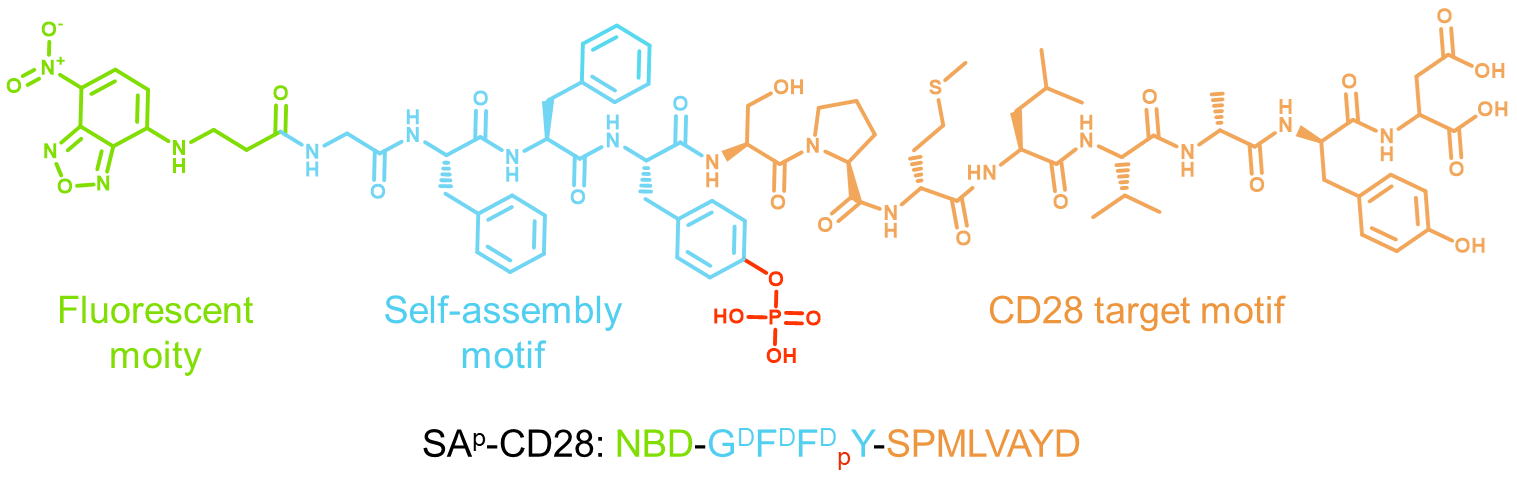


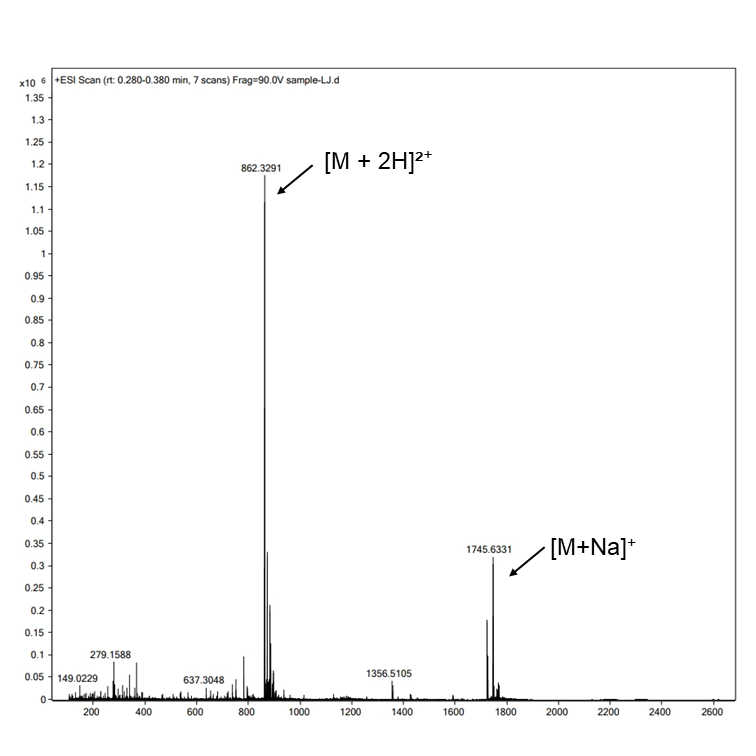


**Figure S1.** Chemical structure and ESI-MS spectra of SA^p^-CD28. [M + 2H] ^2+^_cal._ = 862.3854, [M + 2H] ^2+^_obs._ = 862.3291, [M + Na] ^+^ _cal._ = 1745.7708, [M + Na] ^+^ _obs._ = 1745.6331.


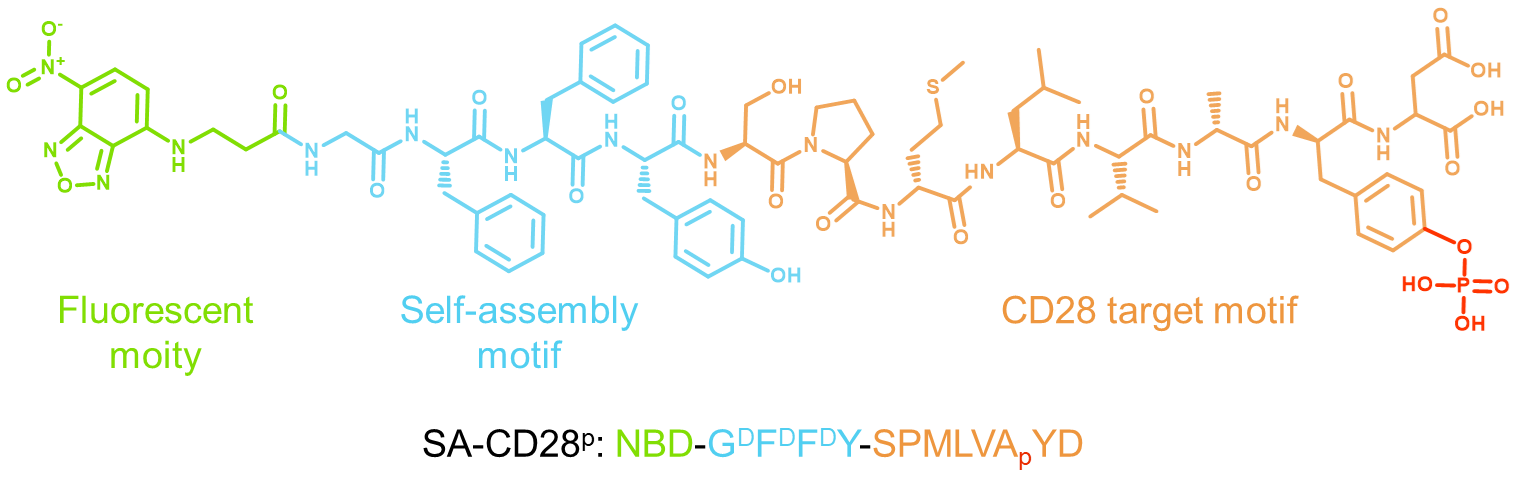


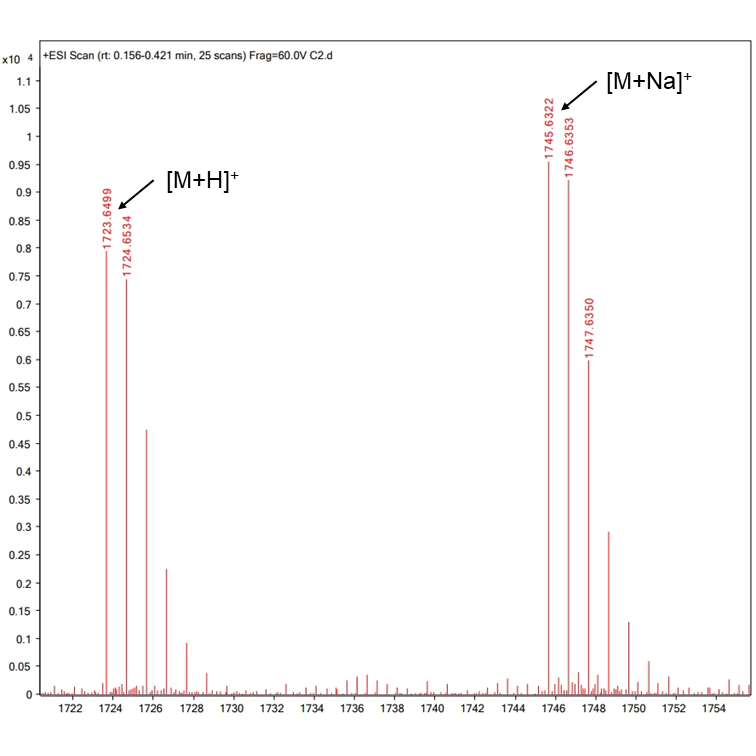


**Figure S2.** **Chemical structure and ESI-MS spectra of** SA-CD28^p^**. [M + H]** ^+^**_cal._ = 1723.7708, [M + H]** ^+^**_obs._ = 1723.6499, [M + Na] ^+^ _cal._ = 1745.7708, [M + Na] ^+^ _obs._ = 1745.6322.**


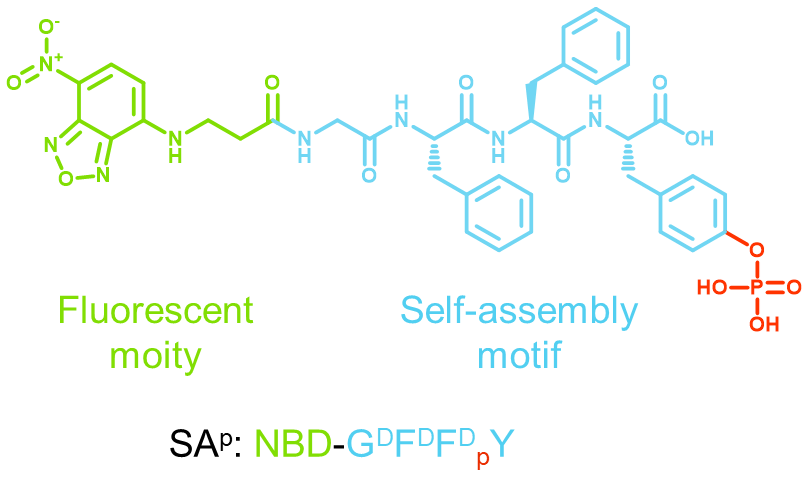


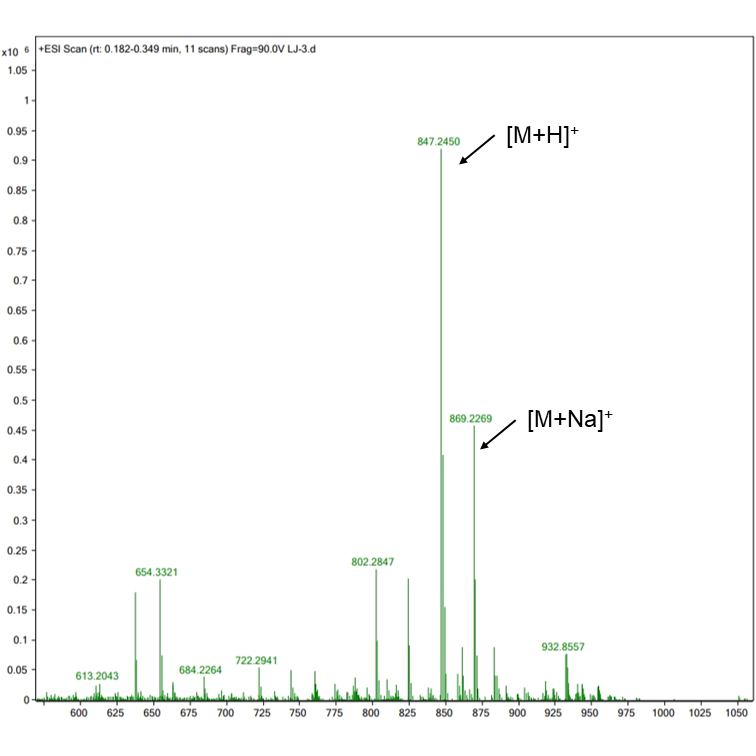


**Figure S3. Chemical structure and ESI-MS spectra of** SA^p^**. [M + H]** ^+^ **_cal._ = 846.7468, [M + H] ^+^ _obs._ = 847.2450, [M + Na] ^+^ _cal._ = 868.7468, [M + Na] ^+^ _obs._ = 869.2269.**

**
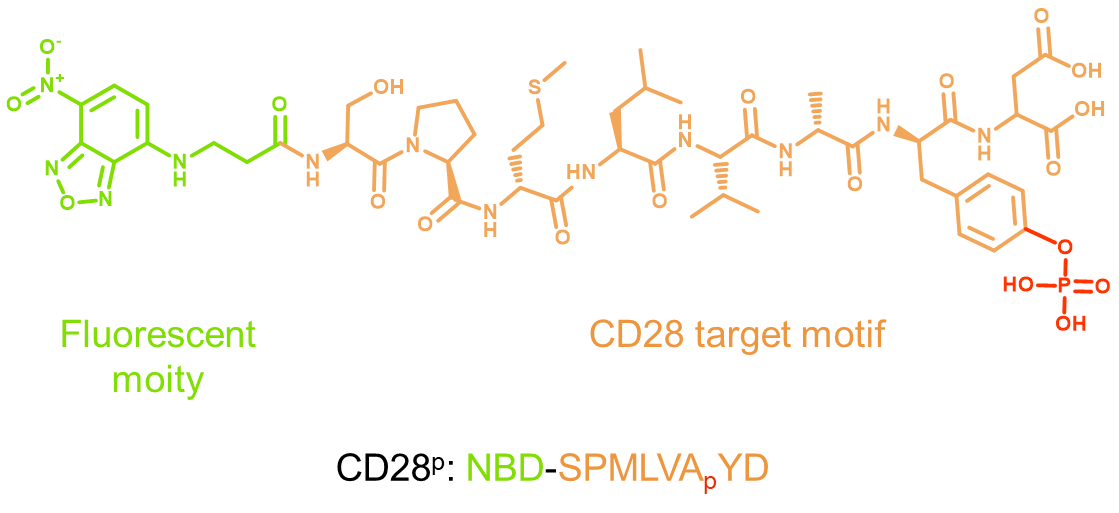
**

**
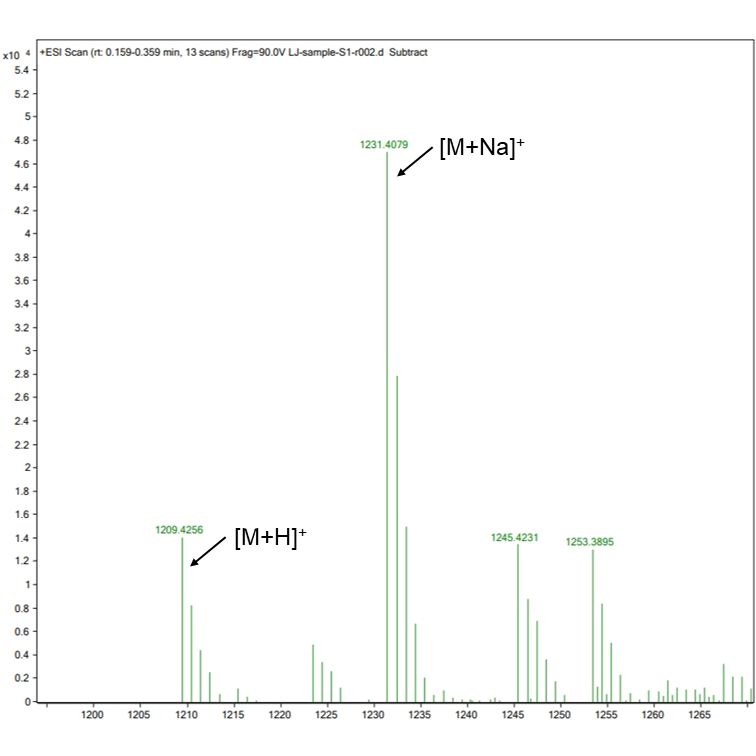
**

**Figure S4. Chemical structure and ESI-MS spectra of** CD28^p^**. [M + H] ^+^ _cal._ = 1209.1888, [M + H] ^+^_obs._ = 1209.4256, [M + Na] ^+^ _cal._ = 1231.1888, [M + Na] ^+^ _obs._ = 1231.4079.**

**
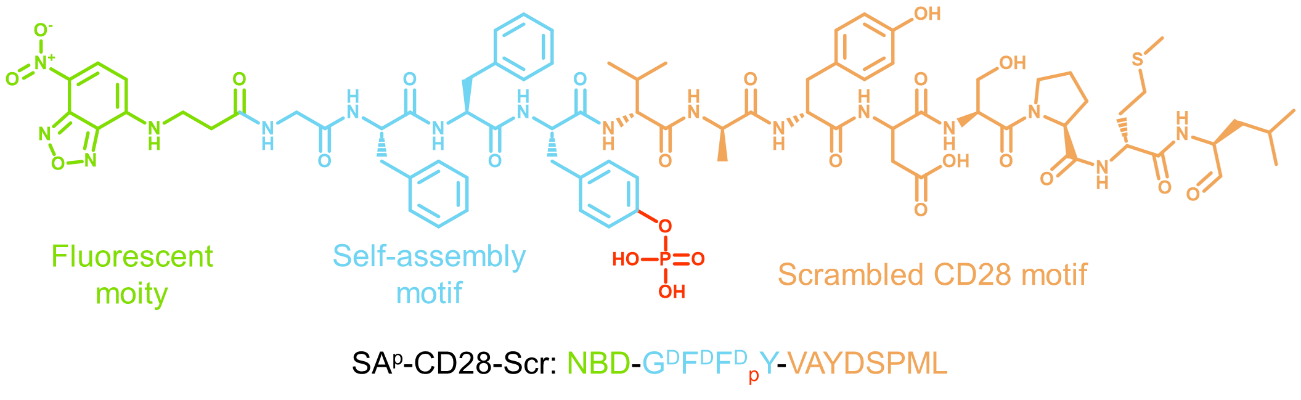
**

**
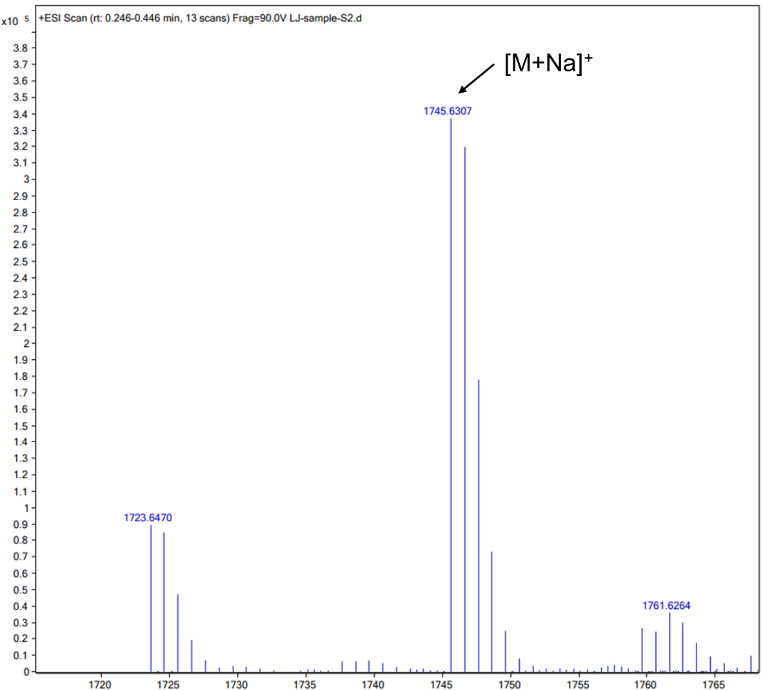
**

**Figure S5. Chemical structure and ESI-MS spectra of** SA^p^-CD28-Scr**. [M + H] ^+^_cal._ = 1723.7708, [M + H] ^+^ _obs._ = 1723.6470, [M + Na] ^+^ _cal._ = 1745.6307.**

**2.2 FTIR and CD Spectroscopic Analysis of Peptide Conformation**

**
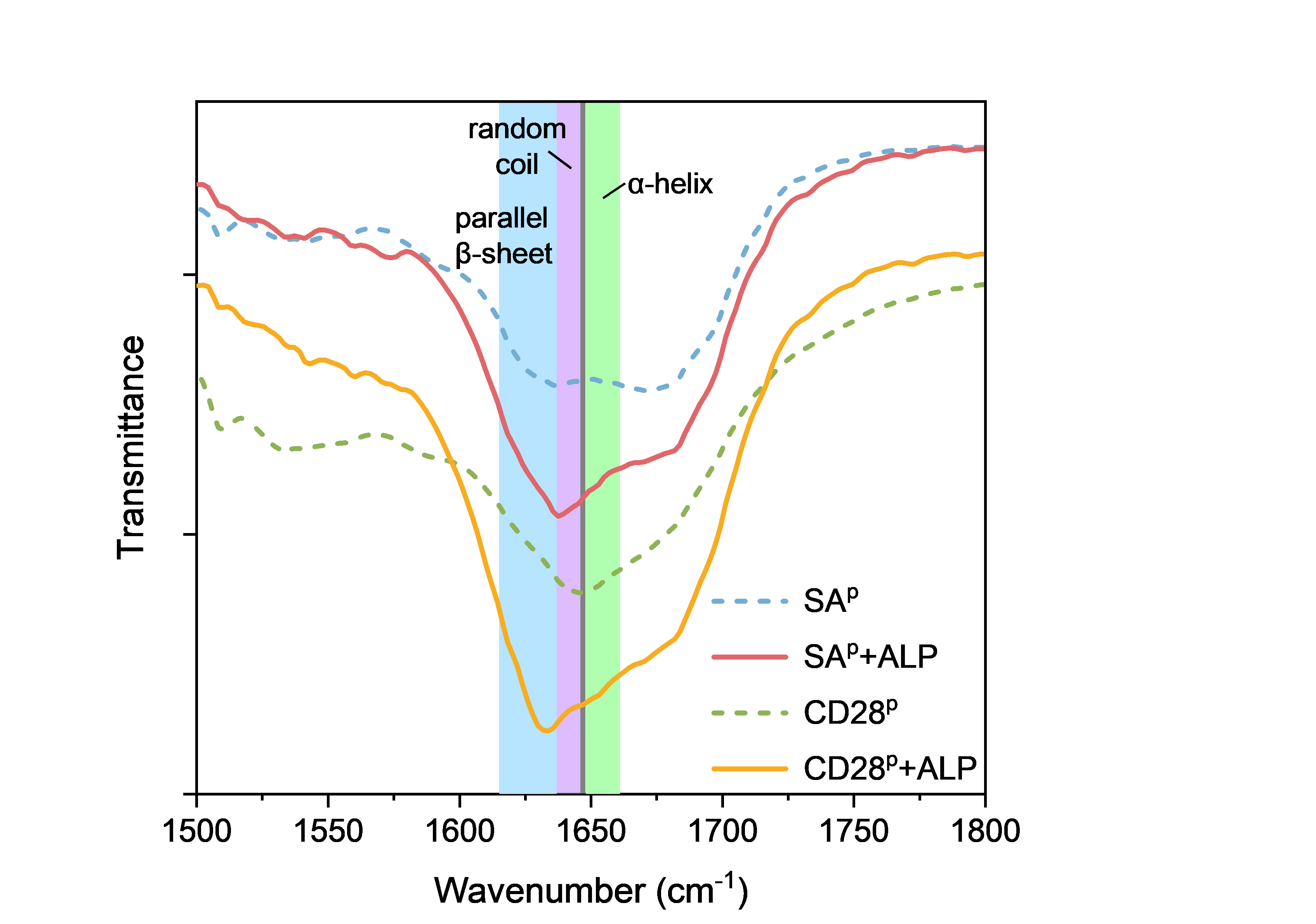
**

**Figure S6. Fourier-transform infrared (FTIR) spectra of** SA^p^ **and** CD28^p^ in the presence or absence of ALP (10 U/mL).


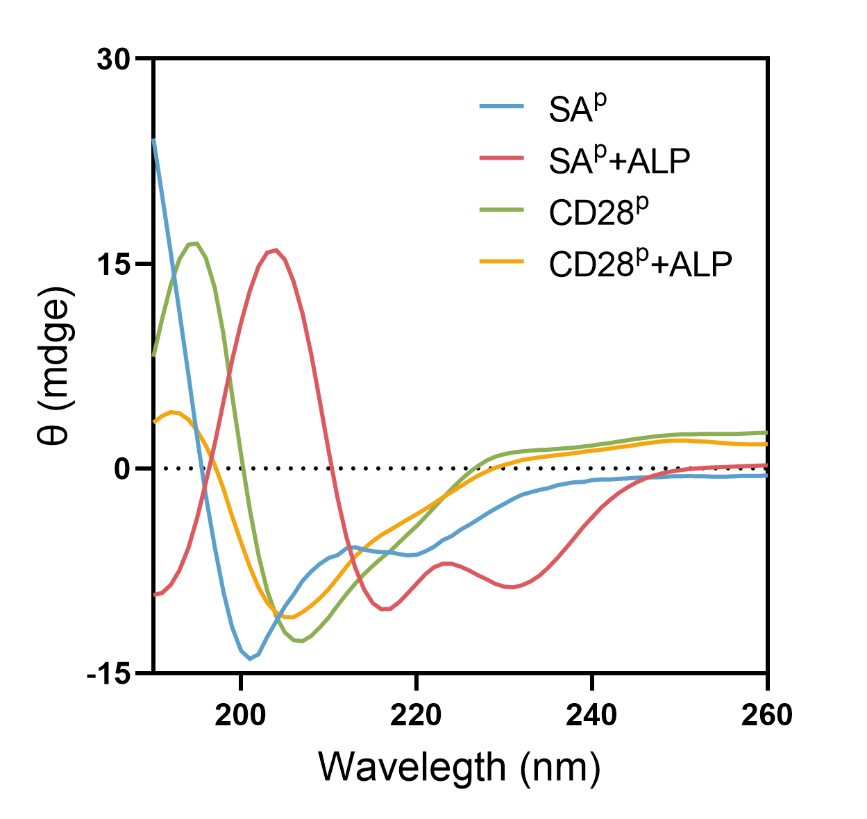


**Figure S7.** **Circular dichroism (CD) spectra of** SA^p^ **and** CD28^p^ in the presence or absence of ALP (10 U/mL).


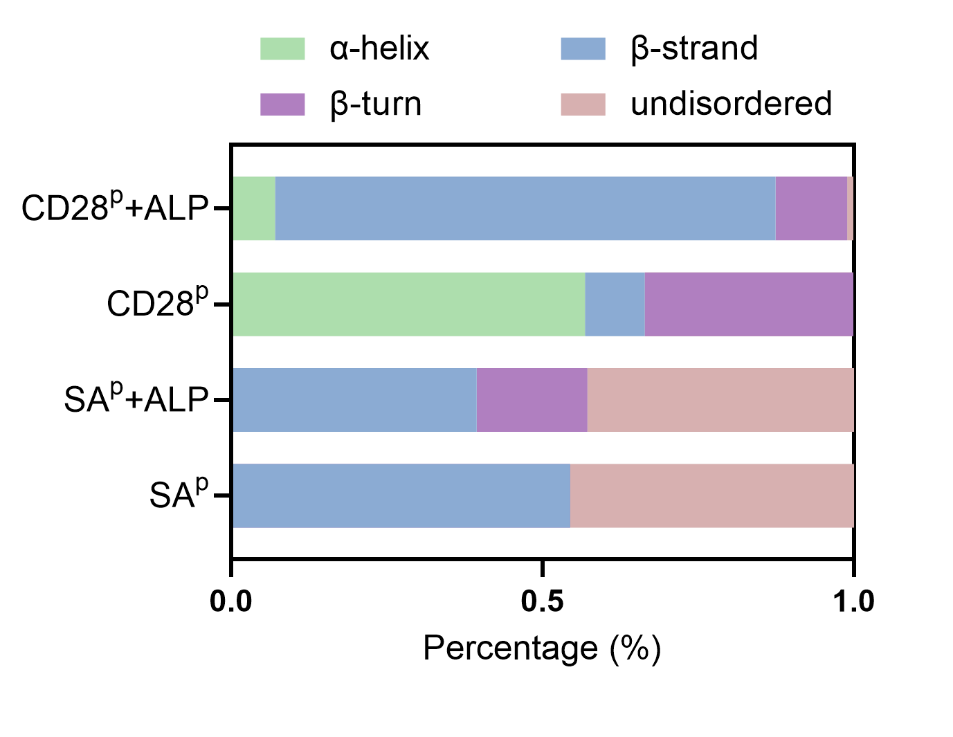


**Figure S8. Secondary structure calculations of** SA^p^ **and** CD28^p^ in the presence or absence of ALP (10 U/mL).

**2.3 Raw MST traces and thermophoresis time window selection**

**
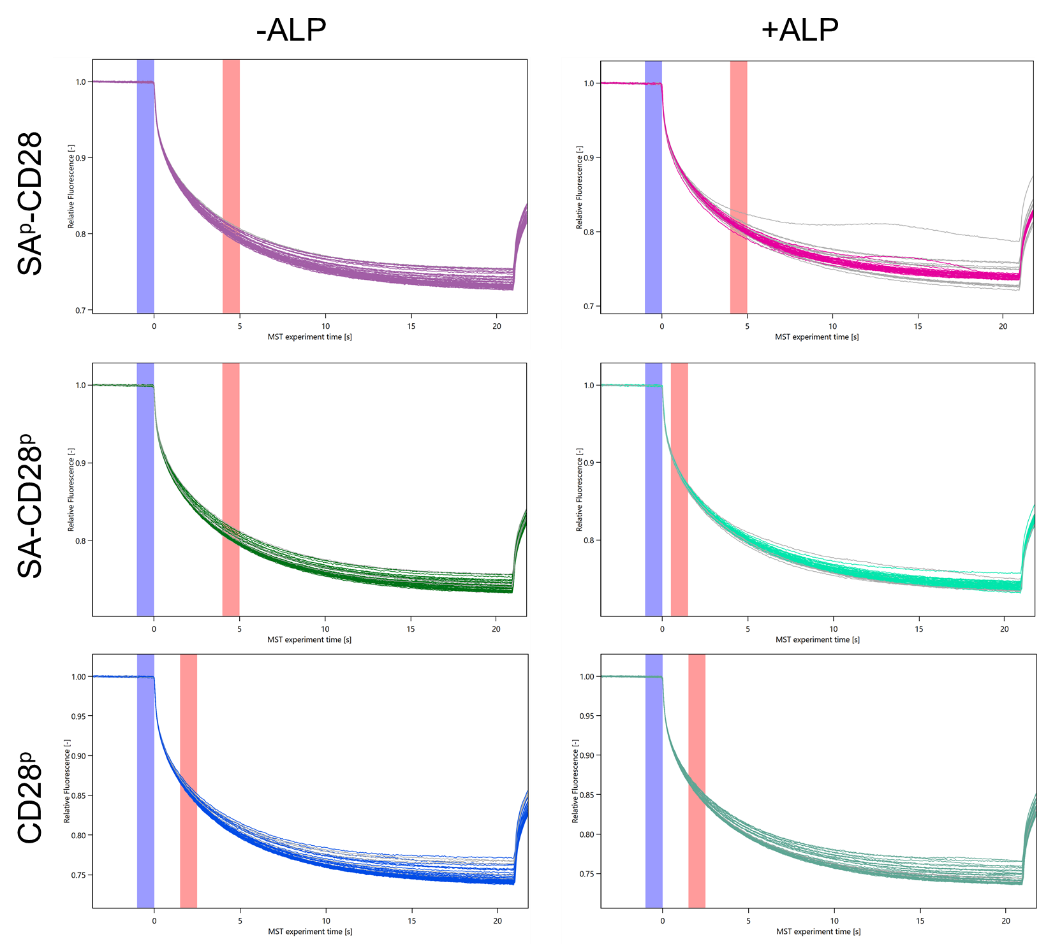
**

**Figure S9. Typical thermophoresis traces for SA^p^-CD28, SA-CD28^p^, and CD28^p^ in the absence (left panels) or presence (right panels) of ALP (10 U/mL). The shaded orange areas indicate the thermophoresis time window selected for K_D_ calculation. Data are presented as mean ± SD (n = 3 independent experiments).**

**2.4 MST Binding Curves of Peptides with and without ALP**

**
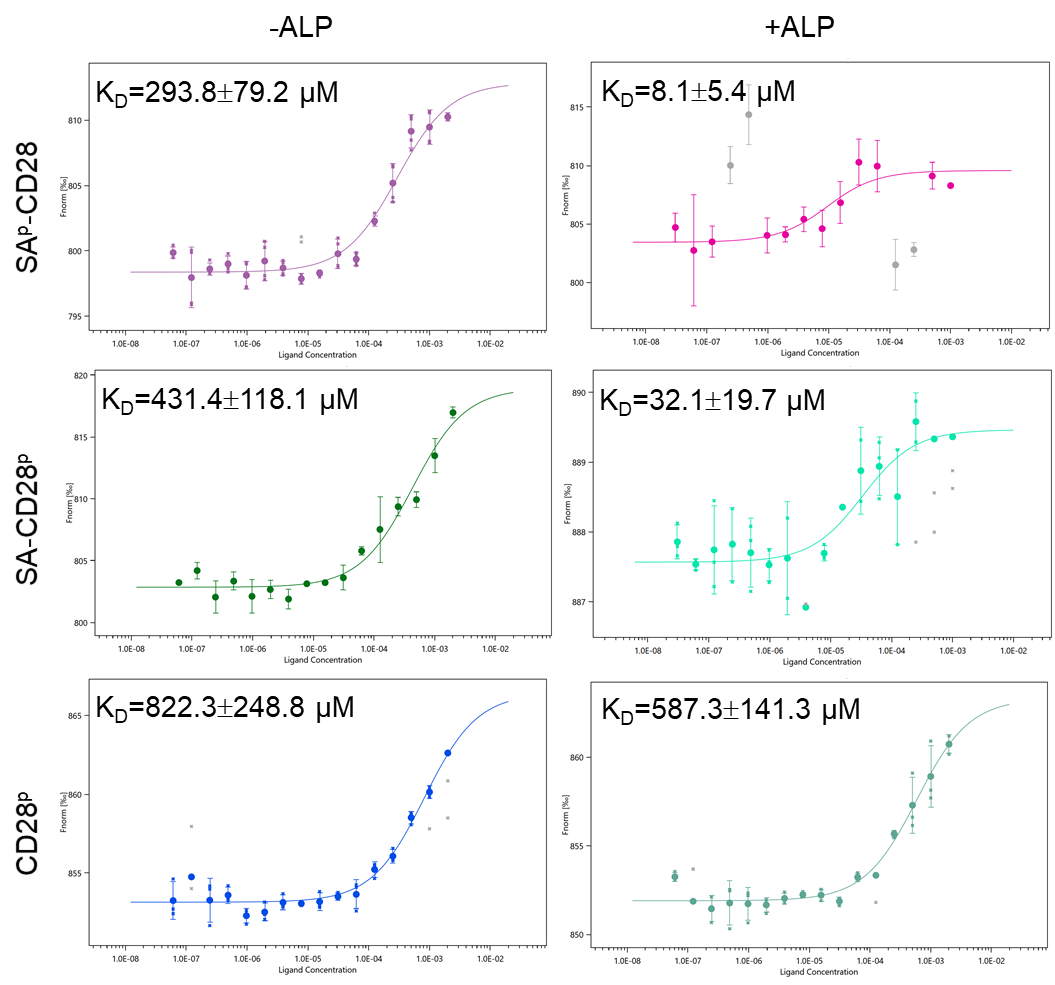
**

**Figure S10. Fitted microscale thermophoresis (MST) curves of** SA^p^-CD28**,** SA-CD28^p^**, and** CD28^p^ **in the absence (left panels) or presence (right panels) of ALP (10 U/mL).** **Data are presented as mean ± SD (n = 3 independent experiments).**

**2.5 Measurement of alkaline phosphatase secretion and CD28 expression levels in human cell lines**


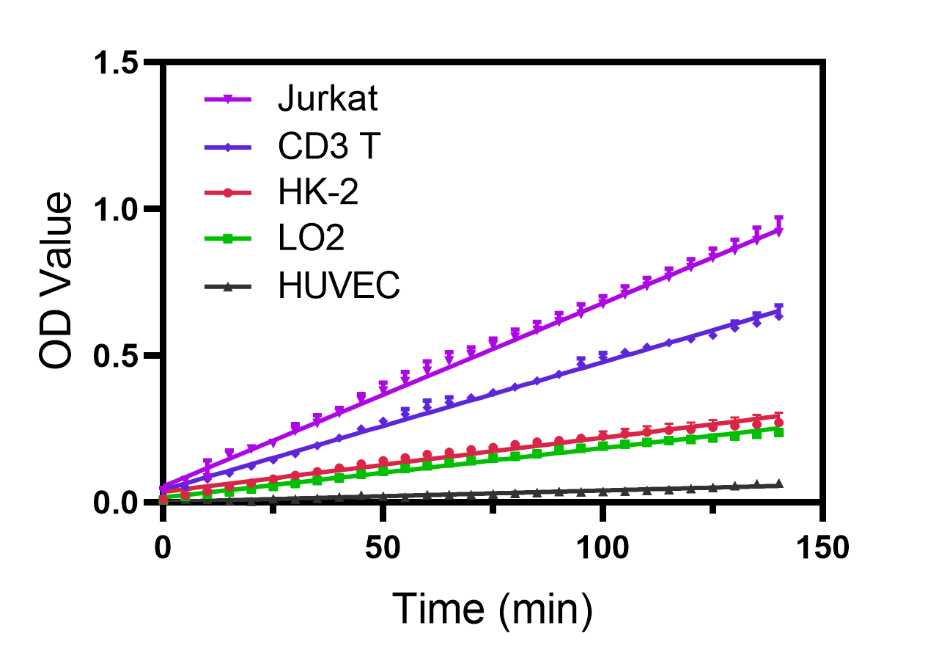


**Figure S11. Measurement of extracellular alkaline phosphatase (ALP) release rate in human cell lines. Data are presented as mean ± SD (n = 3 independent experiments).**

**
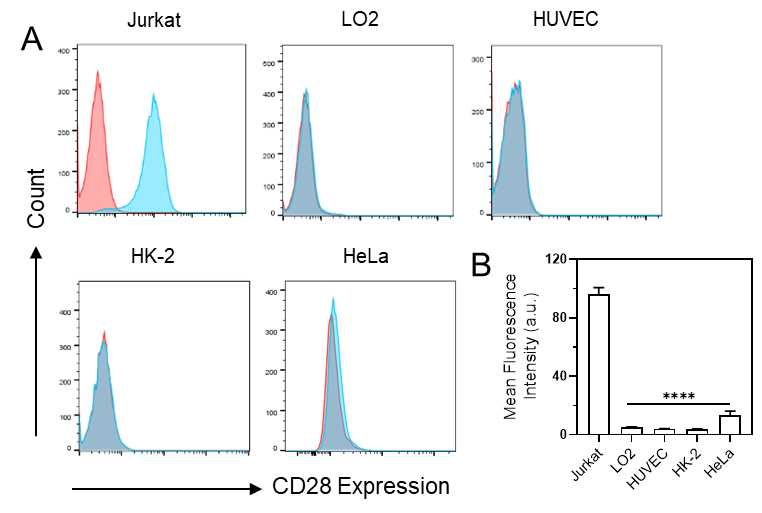
**

**Figure S12. (A) Flow cytometric analysis of surface CD28 expression. (B) Mean fluorescence intensity (MFI) of human cells stained with FITC-labeled anti-CD28 antibody.** Data are presented as mean ± SD (n = 3 independent experiments)**; one-way ANOVA; ****P < 0.0001.**

**2.6 Quantitative Analysis and Imaging of Peptide Uptake in Jurkat Cells**

**
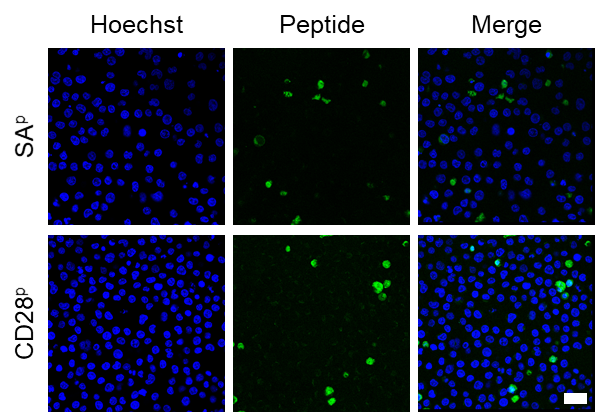
**

**Figure S13. CLSM images of Jurkat cells treated with** SA^p^ **or** CD28^p^**. Scale bar: 25 μm.**

**
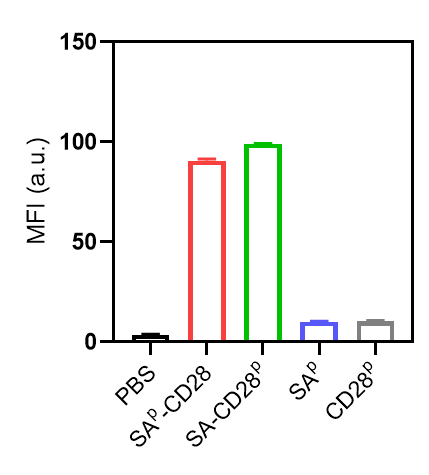
**

**Figure S14. Flow cytometric quantification of NBD mean fluorescence intensity (MFI) in Jurkat cells following peptide treatment.** Data are presented as mean ± SD (n = 3 independent experiments).

**2.7 Lysosomal Colocalization and Escape of SA^p^-CD28**

**
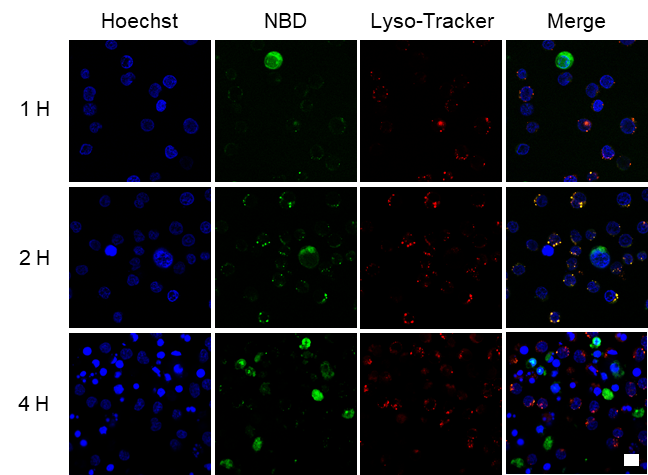
**

**Figure S15. CLSM analysis of lysosomal colocalization and escape of SA^p^-CD28. Scale bar: 10 μm.**

**2.8 Quantitative analysis of the endocytosis mechanism of SA^p^-CD28**

**
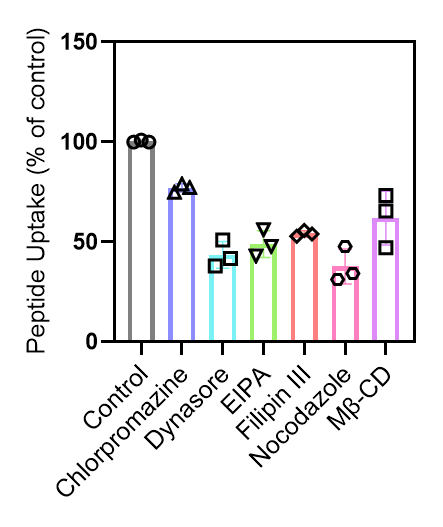
**

**Figure S16. NBD MFI of Jurkat cells pretreated with various endocytosis inhibitors and incubated with** SA^p^-CD28 **for 1 h. Data are presented as mean ± SD (n = 3 independent experiments).**

**2.9 Time-dependent TEM characterization of enzymatic assembly**

**
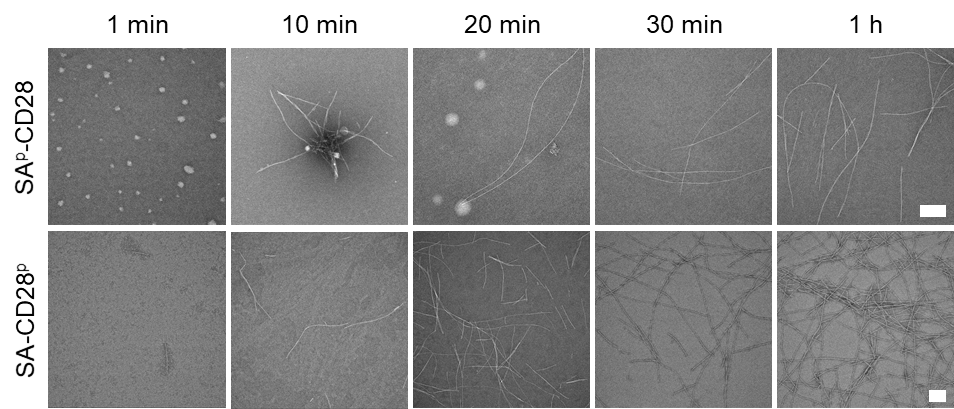
**

**Figure S17. Transmission electron microscopy (TEM) images of** SA^p^-CD28 **and** SA-CD28^p^ **incubated with ALP (10 U/mL) for different time intervals. Scale bar: 200 nm.**

**2.10 Cytotoxicity Assessment of Peptides in LO2 Cells**

**Figure S18. Relative viabilities of** human hepatocyte cell line L-02 (LO2) **incubated with different concentrations of** the four peptides **for 48 h (n = 4).**

**2.11 Quality control of RNA sequencing samples and transcriptomic data**

**
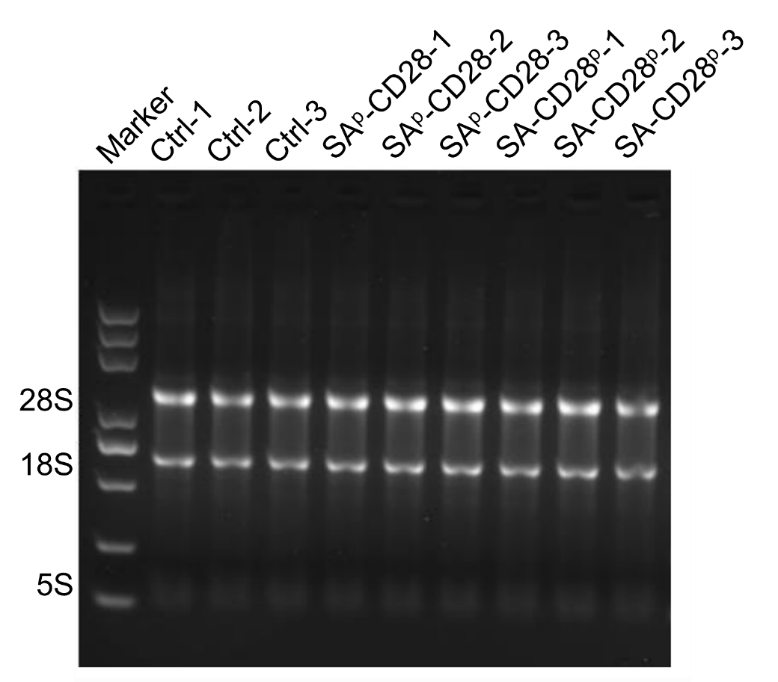
**

**Figure S19. RNA agarose gel electrophoresis. All samples exhibited intact 28S and 18S rRNA bands with approximately 2:1 intensity ratio and no detectable degradation smear.**

| Table S1. Sequencing data quality statistics | | | | |
| --- | --- | --- | --- | --- |
| Sample ID | Q20 (%) | Q30 (%) | GC (%) | Error (%) |
| SA^p^-CD28-1 | 98.23 | 94.67 | 48.78 | 0.02 |
| SA^p^-CD28-2 | 98.3 | 94.5 | 48.31 | 0.01 |
| SA^p^-CD28-3 | 98.25 | 94.76 | 48.73 | 0.03 |
| SA-CD28^p^-1 | 98.37 | 94.72 | 47.98 | 0.01 |
| SA-CD28^p^-2 | 98.24 | 94.69 | 48.25 | 0.02 |
| SA-CD28^p^-3 | 98.38 | 94.75 | 48.13 | 0.01 |
| Ctrl-1 | 98.41 | 94.83 | 48.29 | 0.01 |
| Ctrl-2 | 98.47 | 95.03 | 48.25 | 0.01 |
| Ctrl-3 | 98.16 | 94.08 | 48.47 | 0.01 |

**Notes: Q20 and Q30 represent the percentage of bases with Phred quality scores ≥20 and ≥30, respectively.**

| Table S2. Mapping statistics of clean reads to the reference genome | | | | |
| --- | --- | --- | --- | --- |
| Sample ID | Total reads | Total mapped  reads (%) | Multiple mapped  reads (%) | Uniquely mapped  reads (%) |
| SA^p^-CD28-1 | 57985488 | 97.88 | 2.53 | 95.35 |
| SA^p^-CD28-2 | 53424932 | 97.63 | 2.58 | 95.05 |
| SA^p^-CD28-3 | 51569324 | 97.92 | 2.52 | 95.40 |
| SA-CD28^p^-1 | 45002476 | 97.55 | 2.52 | 95.02 |
| SA-CD28^p^-2 | 44987140 | 97.72 | 2.47 | 95.25 |
| SA-CD28^p^-3 | 56328750 | 97.58 | 2.57 | 95.01 |
| Ctrl-1 | 57168762 | 97.66 | 2.61 | 95.04 |
| Ctrl-2 | 45850312 | 97.69 | 2.57 | 95.12 |
| Ctrl-3 | 44930114 | 97.55 | 2.54 | 95.01 |

**Notes: Clean reads were aligned to the human reference genome using HISAT2.**


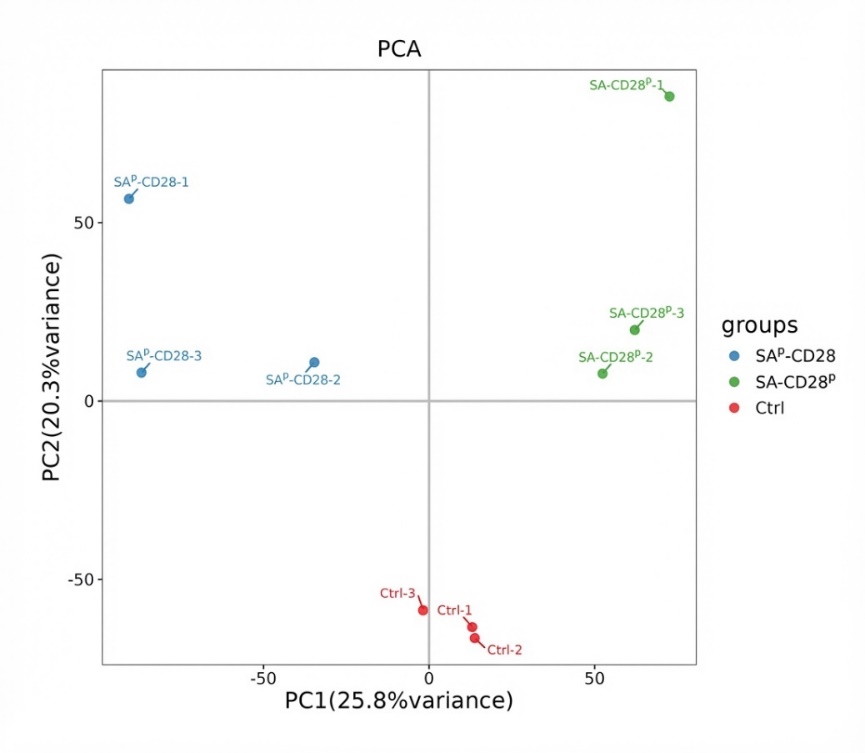


**Figure S20. Principal component analysis (PCA) of transcriptomic profiles showing clear separation among groups and tight clustering within biological replicates (n = 3 per group).**

**2.12 KEGG Pathway Enrichment and Gene Expression Heatmap of SA-CD28^p^-Treated Jurkat Cells**


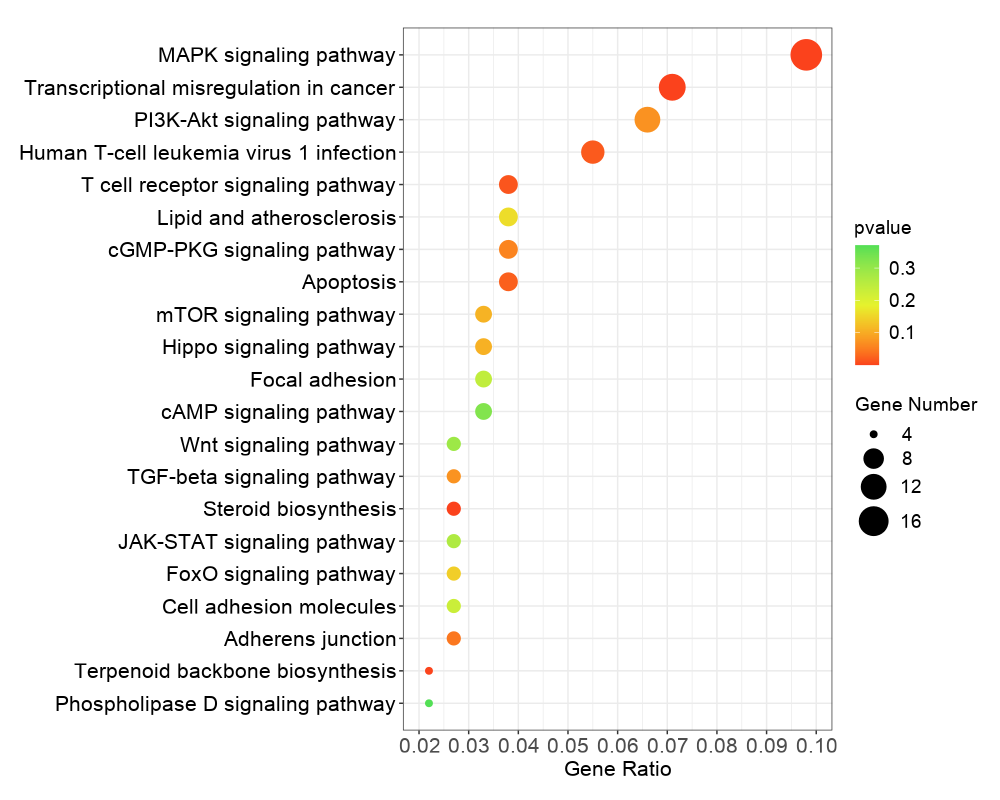


**Figure S21.** KEGG pathway enrichment analysis of differentially regulated pathways in Jurkat cells treated with SA-CD28^p^ compared with the PBS control.

**
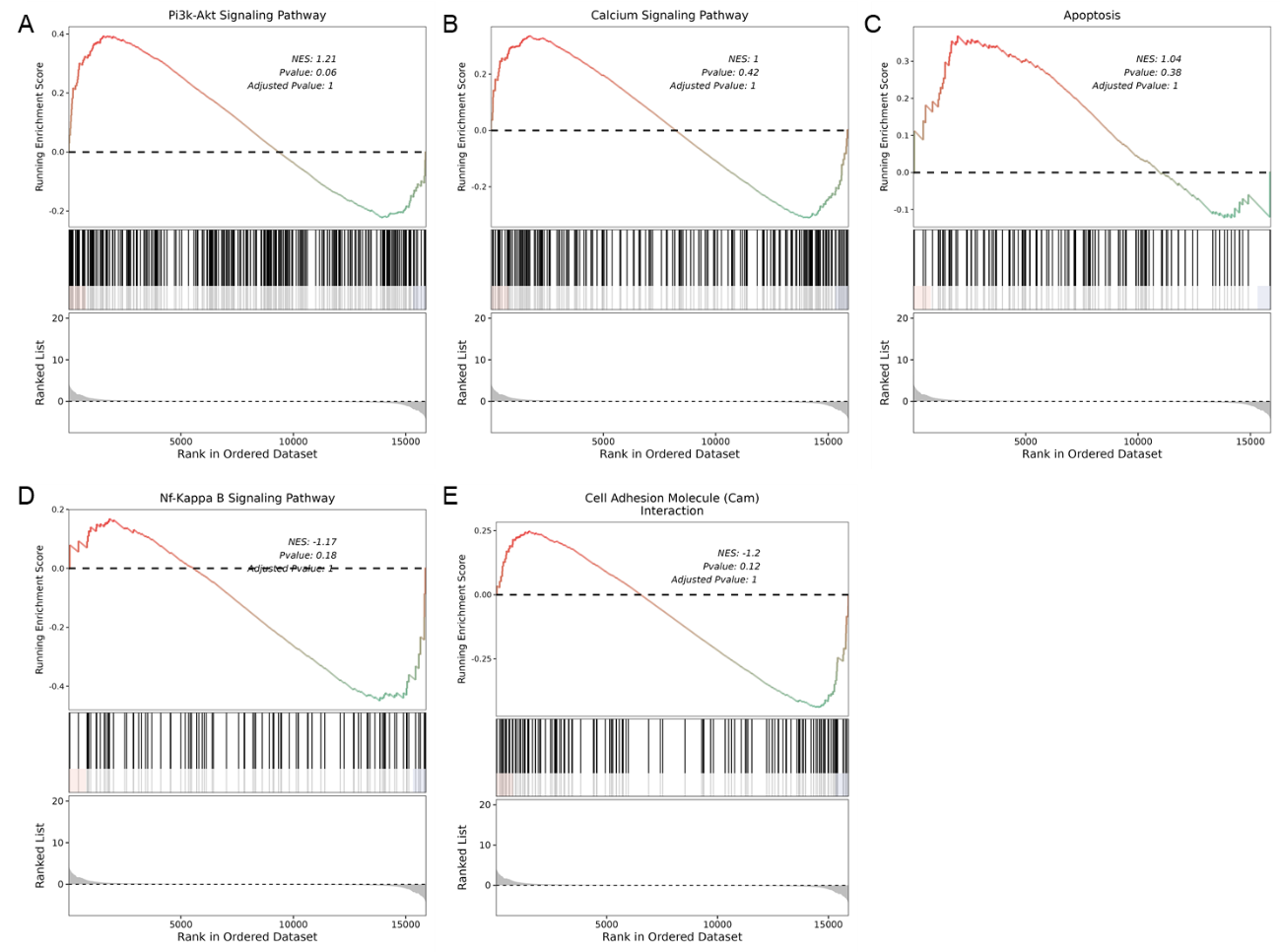
**

**Figure S22.** Gene set enrichment analysis (GSEA) of representative pathways. GSEA plots illustrating the enrichment trends of (A) PI3K-Akt signaling pathway, (B) Calcium signaling pathway, (C) Apoptosis, (D) NF-κB signaling pathway, and (E) Cell adhesion molecules (CAMs) based on the ranked transcriptomic dataset of SA^p^-CD28-treated cells. Statistical parameters including the normalized enrichment score (NES) and nominal P-value, are indicated within each panel. P > 0.05 was considered statistically non-significant but biologically informative for pathway-level trends.

**
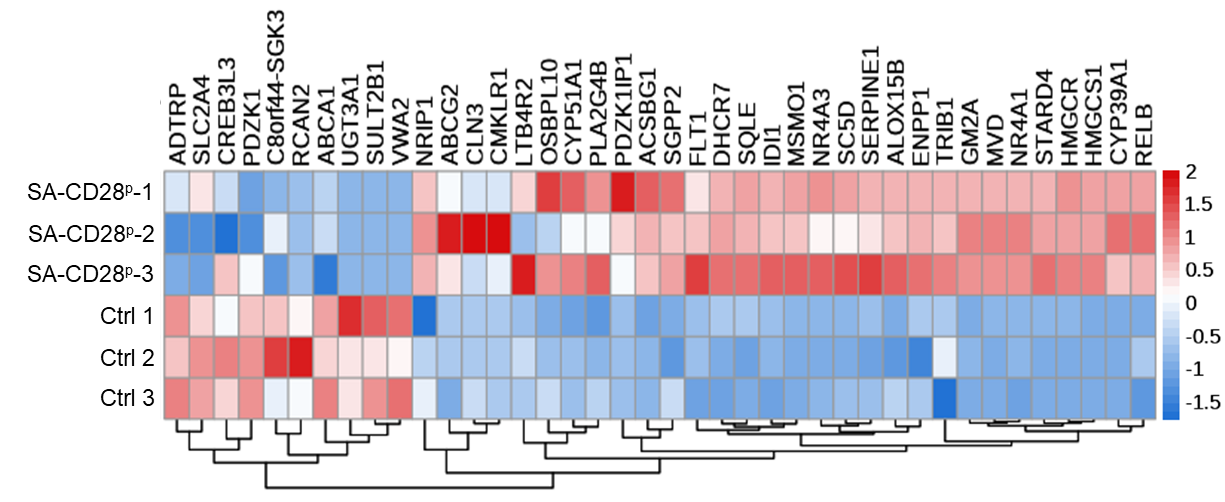
**

**Figure S23.** Heatmap of representative genes associated with apoptosis and nuclear damage in Jurkat cells following SA-CD28^p^ treatment compared with the PBS control.

**2.13** **Control experiments supporting the CD28-dependent activity of SA^p^-CD28**


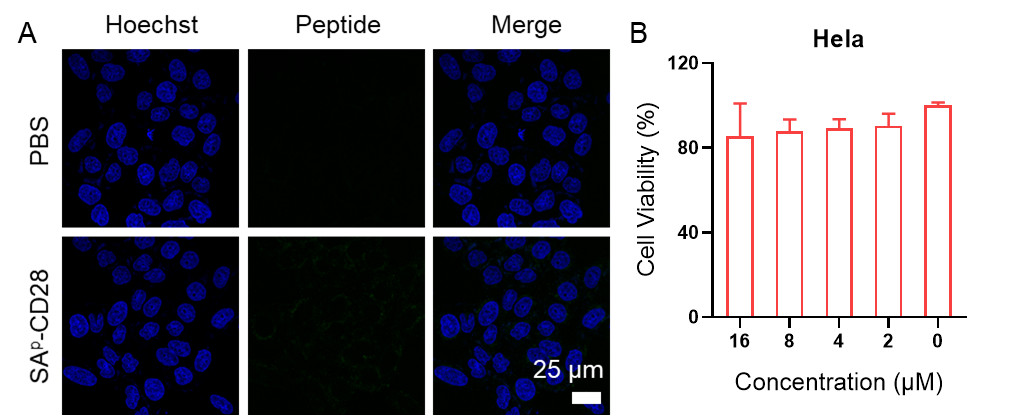


**Figure S24.** Cellular uptake and cytotoxicity of SA^p^-CD28 in HeLa cells. (A) CLSM images of HeLa cells after incubation with SA^p^-CD28. Scale bar: 25 μm. (B) Viability of HeLa cells after 48 h incubation with different concentrations of SA^p^-CD28 (n = 4).


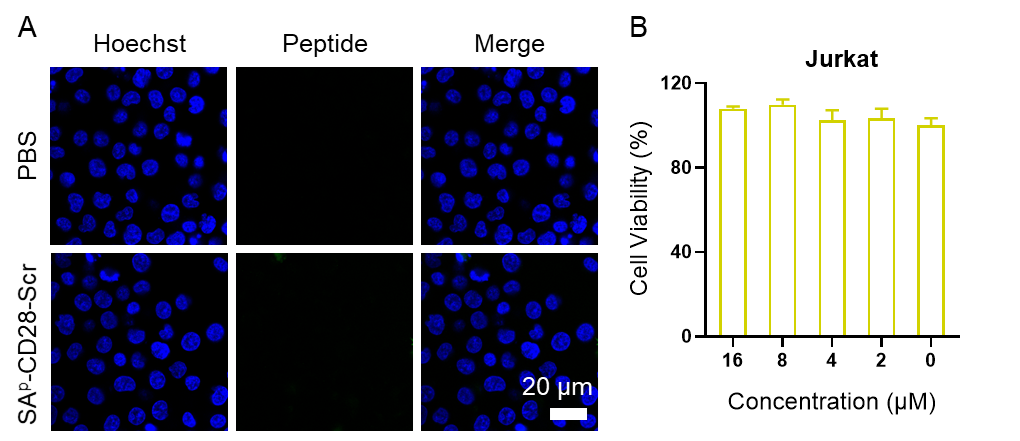


**Figure S25.** **Control experiments using** **SA^p^-CD28-Scr in Jurkat cells.** (A) CLSM images of Jurkat cells treated with the scrambled control peptide SA^p^-CD28-Scr, in which the CD28-targeting sequence SPMLVAYD was rearranged to VAYDSPML. Scale bar: 20 μm. (B) Viability of Jurkat cells after 48 h exposure to different concentrations of SA^p^-CD28-Scr (n = 4).

**2.14 Detection and Quantification of Intracellular ROS Levels**

**
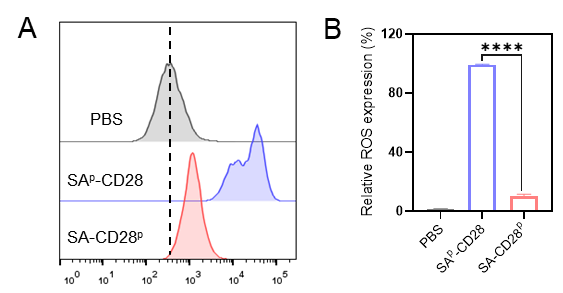
**

**Figure S26.** Flow cytometric analysis of (A) reactive oxygen species (ROS) levels in Jurkat cells and (B) quantification of mean fluorescence intensity. Data are presented as mean ± SD (n = 3 independent experiments); one-way ANOVA; ****P < 0.0001.

**2.15 Histological Examination of Major Organs Across Treatment Groups**

**
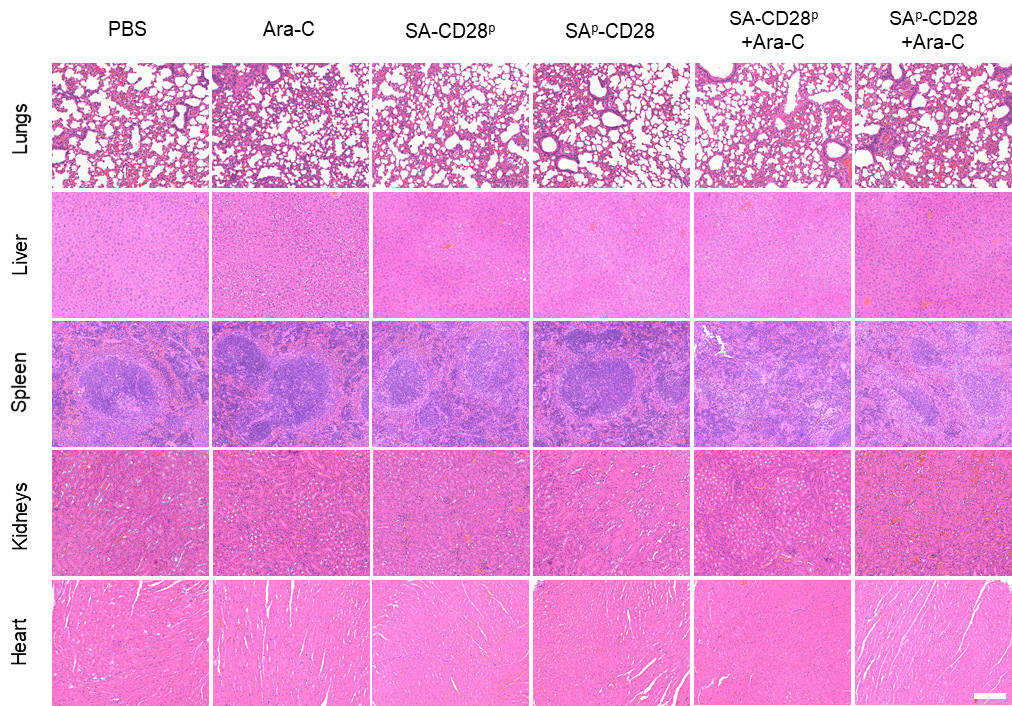
**

**Figure S27.** Representative hematoxylin and eosin (H&E) staining of heart, liver, spleen, lung, and kidney tissues collected at the study endpoint after the indicated treatments. Scale bar: 200 μm.

# References

[1] Y. Ding, D. Zheng, L. Xie, X. Zhang, Z. Zhang, L. Wang, Z.W. Hu, Z. Yang, Enzyme-Instructed Peptide Assembly Favored by Preorganization for Cancer Cell Membrane Engineering, J. Am. Chem. Soc., 145 (2023) 4366-4371.

[2] S. Cao, L.C. da Silva, K. Landfester, Light-Activated Membrane Transport in Polymeric Cell-Mimics, Angew Chem Int Ed Engl, 61 (2022) e202205266.

[3] F. Persson, P. Bingen, T. Staudt, J. Engelhardt, J.O. Tegenfeldt, S.W. Hell, Fluorescence nanoscopy of single DNA molecules by using stimulated emission depletion (STED), Angew Chem Int Ed Engl, 50 (2011) 5581-5583.

[4] C. Lee, S. Peddi, C. Anderson, H. Su, H. Cui, A.L. Epstein, J.A. MacKay, Adaptable antibody Nanoworms designed for non-Hodgkin lymphoma, Biomaterials, 262 (2020).

[5] J. Shi, G. Fichman, J.P. Schneider, Enzymatic Control of the Conformational Landscape of Self-Assembling Peptides, Angew Chem Int Ed Engl, 57 (2018) 11188-11192.

[6] L. Zhang, Z. Wang, J. Das, M. Labib, S. Ahmed, E.H. Sargent, S.O. Kelley, Potential-Responsive Surfaces for Manipulation of Cell Adhesion, Release, and Differentiation, Angew Chem Int Ed Engl, 58 (2019) 14519-14523.

[7] P. Mullen, The Use of Matrigel to Facilitate the Establishment of Human Cancer Cell Lines as Xenografts. In: Langdon, S.P. (eds) Cancer Cell Culture. , Methods in Molecular Medicine™, 88 (2004).

[8] L.N. Tung, S. Song, K.T. Chan, M.Y. Choi, H.Y. Lam, C.M. Chan, Z. Chen, H.K. Wang, H.T. Leung, S. Law, Y. Huang, H. Song, N.P. Lee, Preclinical Study of Novel Curcumin Analogue SSC-5 Using Orthotopic Tumor Xenograft Model for Esophageal Squamous Cell Carcinoma, Cancer Res. Treat., 50 (2018) 1362-1377.
